# Zα and Zβ domains of ADAR1 and ZBP1 bind G-quadruplexes with nanomolar affinities, establishing Zβ as a G-quadruplex-specific domain

**DOI:** 10.1093/nar/gkag419

**Published:** 2026-05-04

**Authors:** Charles W Kroft, Jeffrey B Krall, Michael Warchol, Robb Welty, Alan Herbert, Morkos A Henen, Beat Vögeli

**Affiliations:** Department of Biochemistry and Molecular Genetics, School of Medicine, University of Colorado Anschutz Medical Campus, Aurora, CO 80045, United States; Department of Biochemistry and Molecular Genetics, School of Medicine, University of Colorado Anschutz Medical Campus, Aurora, CO 80045, United States; Department of Biochemistry and Molecular Genetics, School of Medicine, University of Colorado Anschutz Medical Campus, Aurora, CO 80045, United States; Department of Biochemistry and Molecular Genetics, School of Medicine, University of Colorado Anschutz Medical Campus, Aurora, CO 80045, United States; Discovery, InsideOutBio, 42 8th Street, Unit 3412, Charlestown, MA 02129, United States; Department of Biochemistry and Molecular Genetics, School of Medicine, University of Colorado Anschutz Medical Campus, Aurora, CO 80045, United States; Department of Biochemistry and Molecular Genetics, School of Medicine, University of Colorado Anschutz Medical Campus, Aurora, CO 80045, United States

## Abstract

While it is well established that the Zα domains of ADAR1 and ZBP1 proteins bind Z-form-prone nucleic acids (Z-NAs), it has also been shown that the Zα domain of ADAR1 binds DNA G-quadruplexes (GQs). However, no binding partner of the structurally homologous Zβ domain of ADAR1 has been identified to date. Based on AlphaFold and molecular dynamics simulations, it has recently been suggested that Zβ of ADAR1 targets its substrate by recognizing GQs. Here, we provide the first experimental evidence for Zβ binding to select GQ RNA and DNA *in vitro*, with structural specificity and nanomolar affinity. We also demonstrate that the Zα domains of ZBP1 and ADAR1 bind to both DNA and RNA GQs with similar affinity. These findings extend the range of potential functional roles for these proteins and open new hypotheses for testing in cells.

## Graphical Abstract

**Graphical Abstract:**
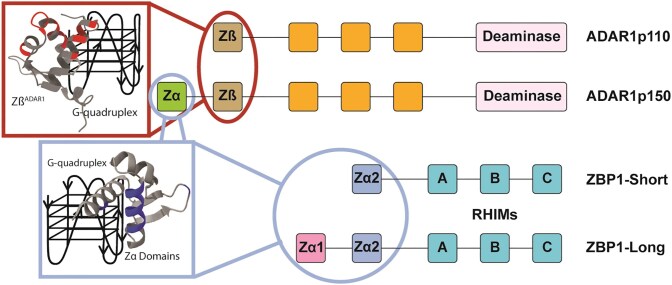

## Introduction

Z-Binding Domains (ZBDs) are known for their ability to bind Z-conformation nucleic acids (Z-NA) and carry the ability to flip specific right-handed helices of nucleic acids (NA) into Z-NA [1–3]. Detection of double-stranded nucleic acids is an important process occurring in all cells, playing a role in pathways such as innate immune response. Interestingly, both double-stranded DNA and RNA (dsDNA and dsRNA) can adopt higher–energy, left-handed Z-conformation [4–6], in addition to their common B- and A-conformations, respectively. Z-DNA is generated through supercoiling during transcription and by the ejection of nucleosomes from DNA. In various disease conditions with dysregulated RNA transcription, such as viral infection, auto-inflammation, and cancer [7, 8], host retroelements are a significant source of Z-RNA formation [9]. In humans, the only two proteins known to contain ZBDs are adenosine deaminase acting on RNA 1 (ADAR1) and Z-DNA binding protein 1 (ZBP1), which regulate immune responses and/or trigger inflammatory cell death [2, 8, 10–14].

**Figure 1.**
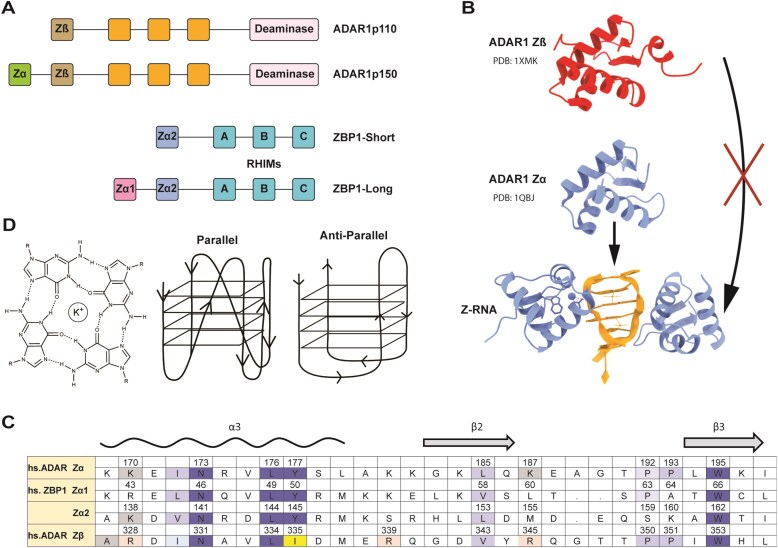
Domain architecture of ADAR1 and ZBP1 and structure of G-quadruplex (GQ). (**A**) Architecture of ADAR1 and ZBP1 isoforms using the single letter IUPAC coding for amino acids. (**B**) Structure of ADAR1 Zα and Zβ, direct interactions with Z-RNA. (**C**) Sequence alignment of human Zα and Zβ. Universally conserved Zα family residues are shaded dark blue, with other conservative substitutions shaded light blue. The Y-to-I variant that abrogates Z-NA binding by Zβ is highlighted in yellow. (**D**) G-tetrad, as well as the structure of anti-parallel and parallel GQs.

ADAR1 is a nucleic acid-binding protein that harbors adenosine-to-inosine editing capability [15, 16]. It is expressed in two primary isoforms; the constitutive ADAR1p110 and the interferon-induced ADAR1p150 protein containing the Zα domain (Fig. 1A) [17]. While ADAR1p150 undergoes nucleocytoplasmic shuffling, ADAR1p110 is predominantly localized to the nucleus [18]. Both ADAR1p150 and ADAR1p110 incorporate a C-terminal deaminase domain, three intervening dsRNA binding domains, and the enigmatic N-terminal Zβ domain [16]. Unlike structurally homologous Zα domains, Zβ cannot flip dsDNA and dsRNA into the Z-form or even bind to pre-stabilized Z-NA (Fig. 1D) [19]. Its binding partners and biological functions have remained unknown. Despite these findings, the Zβ domain is likely functionally important, as its four α-helices are more evolutionarily conserved across species than the respective Zα domain of ADAR1 across species [20, 21]. ADAR1p110 functions to locate and edit self-dsRNA while still in the nucleus, where it is thought to play roles in alternative splicing, microRNA (miRNA) maturation, and recoding of transcripts [22]. The triage of inosine-containing transcripts in the nucleus, mostly of retroelement origin, helps prevent errant immune responses [16, 23, 24].

Both Zα and Zβ are paralogs with distinct evolutionary histories. Both consist of three α-helices and three anti-parallel β-sheets, but Zβ lacks the conserved tyrosine that plays an essential role in Z-NA binding by Zα [20, 25, 26]. The Zα NA binding interface has four conserved residues across all members of the Zα domain [2, 21, 26]. In the Zα domain, N173, Y177, and W195 in the recognition helix (α3) and β-wing are indispensable (UniProt: P55265) for structure-specific interaction with Z-form nucleic acids [25, 26] (Fig. 1C). L176 contributes to the hydrophobic core of the helix.

While ADAR1p110 functions to reduce the level of dsRNA in the cell by promoting inosine-dependent degradation, ADAR1p150 prevents an immune response to endogenous self-dsRNA, most of which are produced from retroelements [27–30]. The recognition of these elements by ADAR1p150 modulates the innate immune response, in part by squelching the activation of inflammatory cell death by ZBP1 [11–14]. The full-length product of ZBP1 is comprised of three RIP-homotypic interaction motifs (RHIMs) and two tandem Zα domains (Zα1 and Zα2) (Fig. 1A) [31, 32]. The RHIM motifs facilitate downstream signaling upon the interaction of ZBP1 with Z-NA, leading to three major cellular responses: apoptosis, necroptosis, and pyroptosis, depending on various cellular conditions [8]. ZBP1 is spliced into two predominant isoforms, ZBP1-Long (ZBP1-L) and ZBP1-Short (ZBPI-S) [33]. In humans, ZBP1-L contains both Zα domains in addition to the RHIMs [31]. On the other hand, ZBP1-S contains only Zα2 and RHIM motifs. ZBP1-S is sufficient for mediating crisis response, whereas ZBP1-L exhibits a different subcellular localization from its shorter counterpart and is more efficient at initiating necroptosis. The findings hint at different roles for the two isoforms, as there are for ADAR1p150 and ADAR1p110 [34]. Overall, ZBP1 and ADAR1 function in a regulatory interplay, wherein ZBP1 senses cytosolic Z-NA and dsRNA to initiate innate immune responses and programmed cell death. At the same time, ADAR1 binds and edits these same nucleic acids to suppress aberrant ZBP1 activation and maintain immune homeostasis.

Various targets for Zα domains have been identified in the past, including inverted-repeat ALU (IR-ALU) short interspersed nuclear elements (SINEs) within the noncoding regions of messenger RNAs (mRNAs) [35, 36]. Interestingly, it has been shown that Zα also binds to the parallel DNA GQs formed in the oncogenic c-Myc promoter, albeit with residues distinct from those used for Z-NA binding [37]. Furthermore, it has been demonstrated that ADAR1p110 localizes to telomeres and modulates R-loop formation in an editing-dependent manner [38]. The telomeric repeat-containing RNA (TERRA) produced from telomeric and subtelomeric regions of the genome can activate ZBP1 when a cell is undergoing replicative stress, initiating a MAVS-mediated innate immune signaling and triggering a telomere-driven crisis response [34]. TERRA-colocalized ZBP1 oligomerizes into filaments on the outer mitochondrial membrane as part of the response [34]. However, these interactions were not characterized at the molecular level.

Of interest is that TERRA DNA sequences form anti-parallel GQs *in vitro* [39, 40]. In GQ structures, four guanines base pair to form a G4 tetrad stabilized by Hoogsteen hydrogen bonding. The tetrads then stack on each other to create a four-stranded GQ helix (Fig. 1D) [41–43]. Sequences prone to form GQ are G-rich. The classical GQ motif is composed of four stretches of two or more guanines, with one or more bases serving as loops to connect the four strands [i.e, (G_3_U_x_)_4_]. The stability of GQ is also affected by the monovalent ions present in the central cavity. For most GQ folds, K^+^ is the preferred ion, while Li^+^ often disrupts the fold [44, 45]. The functions of GQs depend on where they form, but generally, they serve as regulatory and structural elements in key genomic and cellular processes [46, 47].

Recently, we proposed that certain G-rich ALU RNA sequences form GQ structures [48], serving as a binding target of ADAR1 to localize it to that region of the genome during transcription [49]. As with almost all RNAs, the GQ conformation is parallel and more stable than those formed with DNA [50]. *In silico* molecular docking experiments employing AlphaFold3 [51] and molecular dynamics simulations revealed that a binding interface between both Zα and Zβ to both DNA and RNA GQ structures is plausible, potentially providing an answer to the question of Zβ’s cellular role. In particular, it was proposed that GQs localize ADAR1 to splice sites to offset the detrimental mis-splicing of pre-mRNA that arises from SINE element insertions into active genes, while also contributing to transcript recoding [52]. Interestingly, GQs are significantly enriched downstream of ALUs, placing them within the RNA loop formed when an ALU inverted repeat folds to create a dsRNA editing substrate. The effects of ADAR1 on splicing are even observed in mice in which the enzyme is catalytically inactive, consistent with ADAR1 performing other functions beyond dsRNA editing [53].

GQs are also enriched in other repeat elements such as long interspersed repeats and the SINE-VNTR-ALUs composite hominid retrotransposon, as well as in human endogenous retroviruses [52]. Many viruses with GC-rich genomes also harbor GQ-forming sequences [54]. Along with Z-NAs, GQs represent a potential vulnerability that the host can target to modulate such threats.

Given the potential biological importance of the interaction between ADAR1 and GQ to the host, and the previously discovered interaction between Zα and a specific GQ, we seek here to broadly test and characterize the interactions between the ZBDs of ADAR1 and ZBP1 and various GQ structures with differing topologies and loop structures.

## Methods

### Nucleic acid constructs

The following RNA and DNA constructs were tested for binding to the Zα and Zβ domains: (i) TERRA RNA (UUAGGG)_4_ (TERRA-GQ_RNA_), (ii) RNA (UUACCG)_4_ (TERRA-mut_RNA_), (iii) ALU-RNA GGGA-GGGC-GGGA-GGG (ALU-GQ_RNA_), (iv) RNA CCGA-CCGC-CCGA-CCG (ALU-mut_RNA_), (v) RNA (GGGU)_3_-GGG (U-loop-GQ_RNA_), (vi) RNA UUACCG, (vii) RNA UUAGGG, and (viii) DNA (GGGGTTT)_3_-GGGG (TTT-loop-GQ_DNA_). These sequences are displayed in Table 1. TERRA-GQ_RNA_, TERRA-mut_RNA_, ALU-GQ_RNA_, and U-loop-GQ_RNA_ were synthesized by Dharmacon Reagents. TTT-loop-GQ_DNA_, ALU-mut_RNA_, UUAGGG, UUACCG, and TERRA-GQ_RNA_ were synthesized by Integrated DNA Technologies (IDT).

**Table 1.** DNA and RNA constructs used in this study

Sequence	Abbreviation	Confirmed GQ structure	MW (Da)
(UUAGGG)_4_	TERRA-GQ_RNA_	Parallel	7846.75
(UUACCG)_4_	TERRA-mut_RNA_	No GQ	7526.51
GGGA-GGGC-GGGA-GGG	ALU-GQ_RNA_	Parallel	4804.16
CCGA-CCGC-CCGA-CCG	ALU-mut_RNA_	No GQ	4483.92
(GGGU)_3_-GGG	U-loop-GQ_RNA_	Parallel	4999.07
UUAGGG	ssUUAGGG/GQ-UUAGGG	Intermolecular GQ	1915.22
UUACCG	-	No GQ	1835.16
(GGGGTTT)_3_-GGGG	TTT-loop-GQ_DNA_	Antiparallel	7943.20

Each RNA or DNA construct was dissolved in ultra-pure water by diluting to a concentration of 1 mM and heating to 82°C. Concentrations were confirmed before refolding using a Nanodrop 2000 spectrophotometer. The following extinction coefficients were used to calculate the measured concentration (M^−1^cm^−1^): TERRA-GQ_RNA_, 255 800; TERRA-mut_RNA_, 229 400; ALU-GQ_RNA_, 155 600; U-loop-GQ_RNA_, 151 700; TTT-loop-GQ_DNA_, 237 700; ALU-mut_RNA_, 130 900; UUAGGG, 64 100; and UUACCG, 57 500.

To form GQ structures, each RNA or DNA GQ-forming sequence was heat refolded under the following conditions: the NA stock was heated at 82°C for 10 min, diluted to target concentrations in “GQ-promoting buffer” [20 mM NaPi, pH 6.4, 100 mM KCl, 1 mM dithiothreitol (DTT), 0.5 mM ethylenediaminetetraacetic acid (EDTA)], and allowed to cool down at room temperature for at least 20 min. Successful GQ folding was confirmed via CD. ALU-mut_RNA_ and TERRA-mut_RNA_ sequences were treated with the same procedure as GQ constructs prior to measurements to ensure experimental consistency.

For GQ “unfolding,” the NA constructs were diluted to the target concentration in “GQ inhibiting buffer” (20 mM NaPi, pH 6.4, 100 mM LiCl, 1 mM DTT, 0.5 mM EDTA), heated to 82°C, and allowed to cool at room temperature. Additionally, a high NaCl GQ buffer was used, containing 40 mM NaPi, pH 6.4, 100 mM NaCl, 1 mM DTT, and 0.5 mM EDTA.

(CpG)_12_ RNA used for competition assays was synthesized by IDT and diluted to 500 nM for competition assays after heat refolding in GQ-promoting buffer. An extinction coefficient of 2 298 000 m^−1^cm^−1^ was used for RNA quantification.

Examination of nucleic acid–protein phase separation was conducted using a Nanodrop 2000 spectrophotometer. Absorbance at 800 nM was measured as a readout of turbidity, as proteins and nucleic acids exhibit minimal intrinsic absorbance at this wavelength. Increases in absorbance >0.1 were considered to be indicative of phase separation.

### Protein expression and purification

We derived four protein constructs from the N-terminus of *Homo sapiens* ZBP1 (UniProt: Q9H171) and ADAR1 (UniProt: P55265): ADAR1 Zα (139–202) and Zβ (291–366), and ZBP1 Zα1 (1–70) and Zα2 (103–166). The codon-optimized constructs were synthesized by Genscript and subcloned into the pET-28a(+) plasmid using NcoI and BamHI restriction sites, yielding proteins with an N-terminal 6 × His-tag, a thrombin cleavage site, and a T7 tag. The ordered constructs were expressed and purified as described previously [10, 31]. Briefly, the plasmids were transformed and expressed in LOBSTR-BL21(DE3) *Escherichia coli*. The cell cultures for Zα, Zβ, Zα1, and Zα2 were grown in M9 minimal media supplemented with 1 g/l ^15^N ammonium chloride. All cell cultures were grown to an optical density of 0.6 at 600 nm, induced with isopropyl-β -D-1-thiogalactopyranoside (IPTG) at a final concentration of 1 mM, and allowed to express overnight at 21°C. The cell cultures were centrifuged at 4k RPM for 15 min to collect the cell pellets. Pellets were resuspended in the lysis buffer (50 mM Tris–HCl, pH 8.0, 300 mM NaCl, 10 mM imidazole), supplemented with 1 mM DTT, and subjected to sonication for cell disruption. The lysate was clarified by centrifugation at 15k RPM for 30 min to clear cell debris. The clarified supernatant was applied to 5 × 1 ml HisTrap FF columns (Cytiva, Marlborough, MA), washed with 5 column volumes (CVs) of lysis buffer, 5 CVs of wash buffer (50 mM Tris–HCl, pH 8.0, 1 M NaCl, 10 mM imidazole), and eluted in 1 ml fractions over 3 CVs of elution buffer (50 mM Tris–HCl, pH 8.0, 300 mM NaCl, 500 mM imidazole). The eluents were concentrated to 4 ml, and further purification was completed on a size-exclusion HiLoad 16/600 Superdex 75 pg (Cytiva, Marlborough, MA) in the final GQ NMR buffer (20 mM NaPi, 100 mM KCl, 1 mM DTT, 0.5 mM EDTA, pH 6.4). ^15^N-labeled proteins were concentrated using a 3000 MWCO concentrator (Millipore-Sigma, Burlington, MA): Zα to 1.1 mM, Zβ to 2.2 mM, Zα1 to 2.13 mM, and Zα2 to 3.7 mM for later NMR use. Proteins were quantified using a Nanodrop 2000 spectrophotometer. Extinction coefficients used for calculation were: ADAR1 Zα (M^−1^cm^−1^), 6 990; Zβ, 8 480; ZBP1 Zα1, 6 990; and Zα2, 9 970. Giant virus Zα domain (GV2) was purified as described earlier and in previous studies (see IMGM3300021083) [55]. An extinction coefficient of 11 460 M^−1^cm^−1^ was used for quantification.

### Circular dichroism spectroscopy

For circular dichroism (CD) measurements, the DNA GQ and RNA GQ constructs were prepared at a final concentration of 5 µM in GQ-promoting, inhibiting, or high NaCl buffer. All CD measurements were run using a JASCO J-815 CD spectrometer [using Spectra Manager version 2 (JASCO)] in a 0.1 cm quartz cuvette. For GQ confirmation, ellipticity measurements were taken at 35°C between 320 and 240 nM. Intervals of 1 nM were recorded over three accumulations, and averaged to generate CD spectra, at a rate of 100 nM per minute. To determine the melting temperatures of the GQ sequences, the ellipticity was measured at 264 nM, over a range of 20°C–90°C.

To measure protein interactions with GQs, the nucleic acid was diluted to 5 µM in 200 µM buffer. Protein was then added at molar ratios of 1:1, 2:1, 4:1, 8:1, 16:1, 24:1, and 32:1, depending on the construct. Full CD spectra were measured at each titration point.

### NMR spectroscopy

For the 1D ^1^H NMR measurements of GQ sequences, the RNA was prepared at a concentration of 100 µM in GQ-promoting buffer as described previously at a volume of 150 µl, including 10 µl of D_2_O to achieve a concentration of 6.6%. The sample was pipetted into a regular non-Shigemi 3 mm NMR tube. For 2D ^15^N-Heteronuclear Single-Quantum Coherence (HSQC) measurements of ZBDs (ADAR1 Zα and Zβ, ZBP1 Zα1 and Zα2), ^15^N-labeled protein was diluted in GQ NMR buffer to a final concentration of 200 µM at a volume of 150 µl, including 6.6% D_2_O. For binding experiments between nucleic acids and ZBDs, samples were prepared with a final protein concentration of 200 μM, a final RNA concentration of 100 μM, and 6.6% D_2_O at a final volume of 150 µl. Samples were incubated at 42°C for 30 min before measuring. All RNA and protein NMR experiments were performed at 35°C.

NMR spectra were acquired on a BRUKER Avance NEO 600 MHz cryoprobe spectrometer equipped with a 5/3 mm triple resonance cryoprobe (CP2.1 TCI) and sample jet, using TopSpin 4.2.0 (Bruker). All ^1^H carrier frequencies were centered on the water signal. 1D NMR experiments were measured using a spectral width of 25 ppm, 256 scans, and 16 384 complex points. The Watergate scheme was used for water suppression.

The ^15^N-HSQC spectra were acquired with a nonuniform sampling (NUS) scheme generated by the NUS@HMS scheme generator [56] employing 1024 complex data points in the direct dimension and 50% sampling of the original 200 complex points in the indirect ^15^N dimension. The spectral widths were 13.7 and 35 ppm for the ^1^H and ^15^N dimensions, respectively, with a relaxation delay of 1.3 s and 16 scans. All HSQC spectra were assigned using previously published 3D assignments [10, 31]. Data reconstruction of the 2D NUS spectra was performed using the hmsIST software [56]. A solvent subtraction function was applied in the direct dimension. Further data processing and visualization were conducted using NMRPipe/NMRDraw [57]. Spectra analysis was carried out using the CCPNmr Analysis software v2.5.1 [58]. Except for the titration experiments, all HSQC measurements were recorded with a protein concentration of 200 µM and a nucleic acid concentration of 100 µM. GQ melting experiments of ALU and U-loop RNA were performed by collecting 1D ^1^H spectra over the temperature range 35°C–75°C in 10°C increments. Select experiments were repeated in GQ-promoting, inhibiting, and high NaCl buffers. For NMR titration analysis, a series of ^15^N-HSQC experiments was performed with the protein at 200 µM and nucleic acid concentrations ranging from 0 to 200 µM. The chemical shift perturbation (CSP) was calculated using the following equation:


\begin{eqnarray*}
\mathrm{ CSP} = \sqrt {0.2{{{\left( {\Delta {{\ }^{15}}N} \right)}}^2} + {{{\left( {\Delta {{\ }^1}H} \right)}}^2}}.
\end{eqnarray*}

### Isothermal titration calorimetry

Nucleic acid and protein constructs were buffer-matched by dialyzing twice overnight in the same beaker at 4°C into GQ-promoting buffer as described earlier, using 1 kDa cut-off mini dialysis kits (Cytiva). The concentrations after dialysis were measured using a NanoDrop 2000 Spectrophotometer (Thermo Scientific). Protein stocks and nucleic acid stocks were diluted down to a final concentration of 500 and 50 μM, respectively. Binding heat was measured on a Malvern ITC200 instrument [run using ITC 200 version 1.26.1 (Malvern)] at 35°C and a stirring speed of 750 rpm, with 180 s injection delays and a reference power of 9 μcal s^−1^. The titration was performed with 19 consecutive 2 μl injections of 500 µM protein into 50 μM nucleic acid, with an initial injection volume of 0.4 μl. All isothermal titration calorimetry (ITC) thermograms were fitted using Microcal Analysis version 7 SR4 (OriginLab); the details of the fitting are provided in Freyer and Lewis [59]. Fitted ITC parameters are shown in Table 2.

**Table 2.** ITC measured parameters for ADAR1 ZBDs interaction with ALU-GQ_RNA_

Domain	*K*_a_ (M^−1^)	*K*_d_	Δ*H*	Δ*S*	*N* (sites)
ADAR1 Zα	2.35 × 10^6^ ± 5.7 × 10^5^	430 ± 100 nM	−20.36 ± 0.72 kcal/mol	−47.3 cal/mol/deg	1.44 ± 0.03
ADAR1 Zβ	5.54 × 10^5^ ± 9.2 × 10^4^	1810 ± 300 nM	−20.05 ± 1.08 kcal/mol	−40.2 cal/mol/deg	1.12 ± 0.04

### Microscale thermophoresis

Microscale thermophoresis (MST) was performed on a NanoTemper Monolith NT.115 Pico instrument (NanoTemper Technologies GmbH) at 25°C using auto-detect Pico Red at 20%–40% excitation power. His-tagged ADAR1 Zα and His-tagged ADAR1 Zβ were fluorescently labeled by incubating 100 μl of 200 nM protein solution with 100 μl Red-tris-NTA second generation dye (100 nM) for 30 min in GQ-promoting buffer absent of DTT. The reaction mixture was centrifuged for 10 min at 4°C and 15 000 × *g* speed. Twenty nanomolar of the proteins and 16 two-fold dilution series of different nucleic acid constructs were loaded into 16 standard capillaries (NanoTemper Technologies GmbH; highest concentrations were 250 μM for interaction with ALU-mut_RNA_, 15 μM for interaction with ALU-GQ_RNA_, 21 μM for interaction with TERRA-GQ_RNA_, 275 μM for interaction with TERRA-mut_RNA_, and 285 μM for interaction with TTT-loop-GQ_DNA_). The sigmoidal curves obtained were analyzed to extract the *K*_d_ using NanoTemper analysis software. Fitted MST parameters are shown in Table 3.

For the competition assay, Zα or Zβ of ADAR1 was labeled as described earlier and measured at a concentration of 40 nM. (CpG)_12_ was added to a concentration of 500 nM to Zα or Zβ after labeling and incubated at 42°C for 30 min prior to MST measurement. Premium capillaries were used for this experiment. TERRA-GQ_RNA_ was titrated in with a maximum concentration of 10 µM. Curves were fit in NanoTemper analysis software and analyzed via GraphPad Prism for statistical analysis. The one-site-specific binding model was used, and an extra sum of squares F-test comparing the competition assay to previous TERRA-GQ_RNA_ binding was conducted.

For GV2 MST, GV2 was labeled as described earlier. Measurements were conducted with 20 nM protein. ALU-GQ_RNA_ was titrated in with a maximum concentration of 160 µM. The curve was analyzed using NanoTemper analysis software.

### Results

#### G-quadruplexes are formed under *in vitro* conditions

We tested the following RNA and DNA constructs for binding to the Zα and Zβ domains (Table 1): (i) GQ-forming (UUAGGG)_4_ RNA as found in TERRA sequences (TERRA-GQ_RNA_) with a three-base loop, (ii) a similar RNA sequence (UUACCG)_4_ which is mutated such that it no longer forms a GQ (TERRA-mut_RNA_), (iii) GQ-forming GGGA-GGGC-GGGA-GGG RNA as found in ALU elements (ALU-GQ_RNA_) that has a single adenosine or cytosine base connector, (iv) a mutated ALU sequence such that it no longer forms a GQ (CCGA-CCGC-CCGA-CCG) (ALU-mut_RNA_), (v) (GGGU)_3_-GGG forming an RNA GQ with a single uridine in the loops (U-loop-GQ_RNA_), (vi) UUACCG as a single-stranded RNA (ssRNA) control, and (vii) (GGGGTTT)_3_-GGGG forming a DNA-GQ (TTT-loop-GQ_DNA_). DNA and RNA constructs are described in Table 1. Prior to conducting binding experiments (see next section), we determined if these nucleic acid constructs form GQ under our *in vitro* conditions.

##### Circular dichroism confirms the fold of DNA and RNA constructs

TERRA-GQ_RNA_, U-loop-GQ_RNA_, ALU-GQ_RNA_, and TTT-loop-GQ_DNA_ assume parallel (RNA constructs) and anti-parallel (DNA construct) GQ structures in GQ-promoting buffer as expected (Supplementary Figs S1A and B). The anticipated spectral characteristics of various nucleic acid structures are listed in Supplementary Table S1 [60, 61]. CD also confirmed that the mutated RNA sequences TERRA-mut_RNA_ and ALU-mut_RNA_ do not adopt a GQ structure, or any alternative structure such as an i-motif (Supplementary Fig. S1A).

##### RNA G-quadruplexes are stable to high temperatures

We examined the stability of GQ-forming RNA constructs to further confirm successful folding, as stable GQs are known to have high melting temperature [44, 62]. We also confirmed that the predominant buffer ion affects GQ stability in the expected manner [44, 45]. In agreement with previous literature, ALU-GQ_RNA_, U-loop-GQ_RNA_, and TERRA-GQ_RNA_ melt at high temperatures in K^+^ buffer, followed by Na^+^, with Li^+^ being the least stabilizing (Supplementary Fig. S1C). In comparison, we observed that the TERRA-mut_RNA_ construct had a lower melting temperature in both K^+^ and Li^+^ than any other examined RNA construct (Supplementary Fig. S1C). This indicates successful folding of GQ constructs, and a lack thereof with mutant sequences, enabling us to use ion dependence to refine characterization of the NA interactions with ZBDs and interrogate binding under different buffer conditions.

##### 1D NMR further confirms G-quadruplex structure

Using 1D ^1^H NMR, we further interrogated the fold of GQs by tracking the imino peaks in the 10–12 ppm range, characteristic of a GQ structure [44]. In agreement with the CD spectra, TERRA-GQ_RNA_ but not TERRA-mut_RNA_ showed imino peaks indicative of GQ structure (Supplementary Fig. S1D), while ALU-GQ_RNA_ and U-loop-GQ_RNA_ showed imino GQ peaks that responded to altering buffer conditions in a manner consistent with the melting experiments (Supplementary Fig. S1E). We also confirmed that neither UUACCG nor UUAGGG at low concentration could form a GQ, whereas UUAGGG at high concentration did (see below, Supplementary Fig. S14B). NMR visualization of the effect of temperature on GQ structure in various buffer conditions also agreed with previous measurements (Supplementary Fig. S2).

#### Zα and Zβ domains interact with G-quadruplexes

To test the binding of the Zα and Zβ domains to the various GQ-forming RNA and DNA constructs, we used 2D HSQC NMR, which provides a distinct peak for each ^1^H-^15^N bonded pair in the ZBDs at its respective ^1^H and ^15^N resonances. When the nearby binding of nucleic acids alters the local chemical environment, peaks may shift their frequency positions. In addition, decreasing intensities indicate either a slowed-down tumbling caused by increased complex size and/or by exchange (binding and unbinding) on a micro- to millisecond timescale. The small size of ZBDs allows for strong signal via NMR (ADAR1 Zα, 9.3 kDa; Zβ, 11.0 kDa; ZBP1 Zα1, 9.7 kDa; Zα2, 9.6 kDa). Large changes in peak intensity can therefore be attributed to either binding or large complex formation.

##### Zα and Zβ domains interact with TTT-loop-GQ_DNA_

It has been previously reported that Zα of ADAR1 interacts with a DNA GQ, namely the oncogenic c-Myc promoter parallel GQ [37]. We validated the interaction between Zα and DNA GQs with our anti-parallel TTT-loop-GQ_DNA_. Subsequently, we extended the assays to Zα1 and Zα2 of ZBP1 and Zβ of ADAR1.

**Figure 2.**
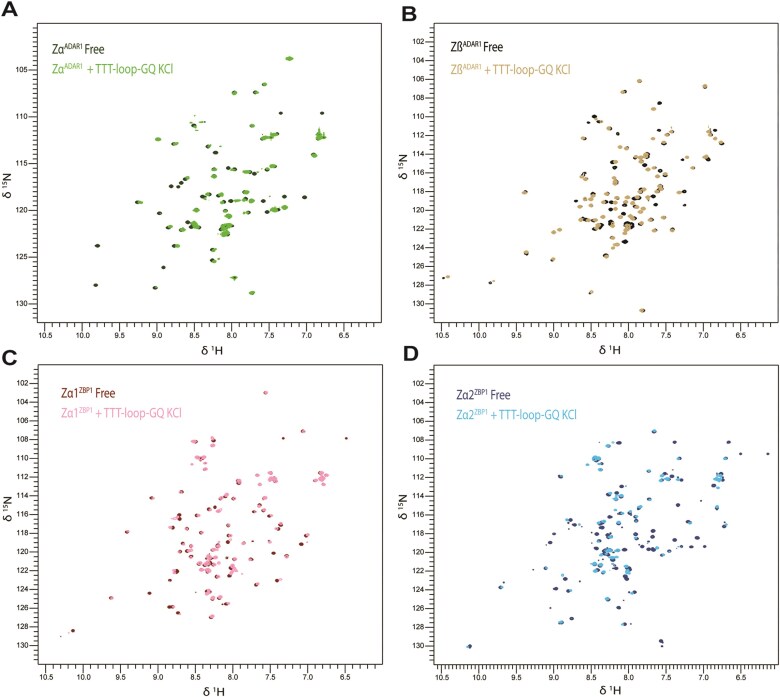
Zα and Zβ domains interact with TTT-loop-GQ_DNA_. Overlaid 2D HSQC spectra of free protein and TTT-loop-GQ_DNA_ in the presence of (**A**) Zα of ADAR, (**B**) Zβ of ADAR, (**C**) Zα1 of ZBP1, and (**D**) Zα2 of ZBP1.

We collected 2D HSQC spectra of both the free and bound proteins to compare binding. Each ZBD-TTT-loop-GQ_DNA_ HSQC binding measurement (and each subsequent HSQC binding experiment) was conducted at a 2:1 protein–NA ratio. Upon addition of the antiparallel GQ DNA to Zα, we observed peak shifts indicative of binding, extending the previous report of an interaction between Zα and a parallel DNA GQ (Fig. 2A).

We then examined whether other ZBDs possessed the same capabilities, especially Zβ, whose binding partner was previously unknown. We repeated the binding experiment for Zβ, and for ZBP1 Zα1, and Zα2. All ZBDs interacted with the TTT-loop-GQ_DNA_, showing peak shifts upon addition of the GQ. Zβ had larger shift changes than any Zα domain (see later CSP analyses, section 4.4), suggesting potentially tighter binding (Fig. 2B). In contrast, Zα1 had smaller peak shifts than the other ZBDs (Fig. 2C and Supplementary Fig. S3E), while Zα2 demonstrated similar peak shifts as did Zβ (Fig. 2D and Supplementary Fig. S3F).

Peak intensities were also affected by binding, as visualized using peak height ratio plots (bound/free) (Supplementary Fig. S3A–D). Zα2 displayed the most significant peak height reduction, followed by Zα and Zβ, which were similar, and Zα1 with the smallest reduction. Altogether, these results display binding interactions of all ZBDs with TTT-loop-GQ_DNA_. The different affinities/binding modes are consistent with the different roles proposed for ZBP1 and ADAR1 isoforms.

##### Zα and Zβ domains interact with U-loop-GQ_RNA_

As we established that each of the Zα (ADAR1 and ZBP1) and Zβ domains interacts with the DNA-GQ construct, we next tested if they also interact with RNA GQs. As the simplest RNA structure, we first probed the interactions of ZBDs with the parallel (GGGU)_3_-GGG, whose loops consist of single U nucleotides (U-loop-GQ_RNA_). Upon addition of U-loop-GQ_RNA_ to each ZBD, we observed varying levels of precipitation/phase separation, which decreased upon incubation at 42°C. Given the consistently observed homogeneity and reversibility, it appears that all ZBDs are phase-separating with U-loop-GQ_RNA_. Zα of ADAR1 and Zα2 of ZBP1 experienced the highest density of condensate formation, Zα1 of ZBP1 intermediate, and Zβ the least.

**Figure 3.**
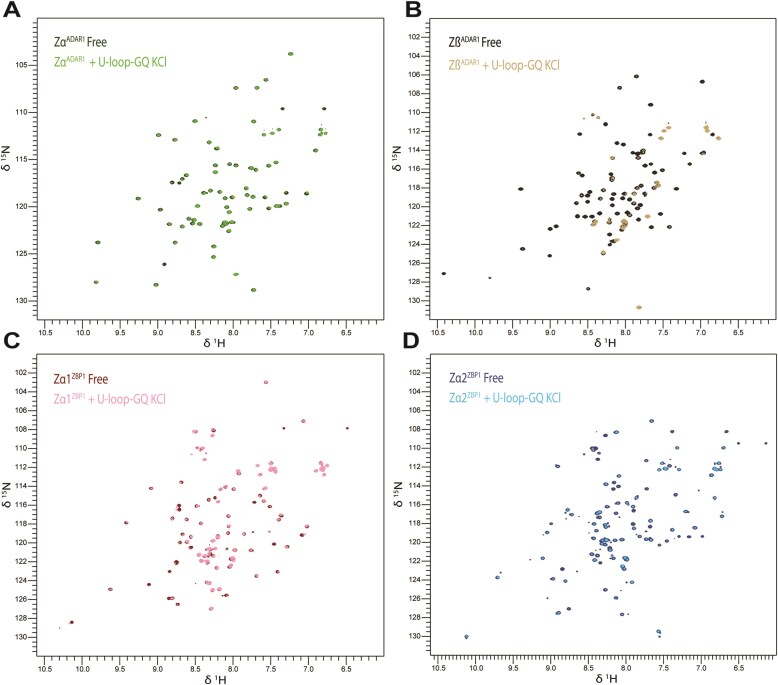
Zα and Zβ domains interact with U-loop-GQ_RNA_. Overlaid 2D HSQC spectra of free protein and U-loop-GQ_RNA_ in the presence of (**A**) Zα of ADAR1, (**B**) Zβ of ADAR1, (**C**) Zα1 of ZBP1, and (**D**) Zα2 of ZBP1.

Using HSQC to visualize binding interaction, we saw that upon addition of the U-loop-GQ_RNA_, Zα underwent only slight peak shifts and no visible peak disappearance (Fig. 3A). While this could indicate that Zα does not strongly interact with U-loop-GQ, the observed liquid–liquid phase separation (LLPS) shows that an interaction is indeed occurring. However, the large protein/RNA complexes so formed prevented HSQC visualization of shifts.

In contrast, upon addition of the U-loop-GQ_RNA_, Zβ displayed significant peak disappearance, with only minor peak shifting (Fig. 3B). These changes indicate that Zβ interacts extensively with the U-loop-GQ RNA in a manner that differs from Zα engagement.

Similarly, Zα1 exhibited significant peak disappearance and reduced peak height, indicative of binding to U-loop-GQ_RNA_. Likewise, Zα1 induced only minor peak shifts (Fig. 3C). These results are similar to the spectrum for Zβ. Intriguingly, both Zβ and Zα1 showed less propensity to form condensates than Zα or Zα2.

For Zα2, we observed similar levels of LLPS as Zα. As expected, we observed an HSQC spectrum with only minor peak losses and minimal shifts (Fig. 3D). This result is again likely due to complex formation, which obscured the visualization of shifts.

We expected to see a direct correlation between the peak height reductions and degree of LLPS; intriguingly, though, we saw the opposite trend, with Zα and Zα2 showing the least peak height reduction relative to free protein, followed by Zα1, and finally Zβ with the greatest degree of peak reduction (Supplementary Fig. S4). This could be explained by LLPS, which sequesters nucleic acids, leaving mainly unbound protein in solution. In the absence of phase separation, a higher fraction of protein and nucleic acid remains in a free state, allowing visualization of their interactions. Thus, peak height reductions appear to reflect both the fraction of nucleic acid interacting with protein and the strength of binding, while also being limited by LLPS.

Overall, these HSQC spectra indicate clear binding interactions between Zβ and Zα1 with the U-loop-GQ_RNA_, as both spectra displayed extensive peak disappearances. While we did not observe peak shifts for ADAR1 Zα and ZBP1 Zα2, we did observe significant reductions in peak intensity for both, but less pronounced than those for ADAR1 Zβ and ZBP1 Zα1.

##### Zα and Zβ domains interact with ALU-GQ_RNA_

Given that each ZBD binds to a minimal RNA parallel GQ, we next tested the interactions with a more biologically relevant construct, ALU-GQ_RNA_, which has loops containing a single adenosine or cytosine. As ADAR1 is known to localize to regions of the genome replete with ALU SINE repeats, it provided an appealing motif to study. Notably, our recent molecular dynamics study proposed that both Zα and Zβ could bind to a GQ of a specific RNA element found in ALU [49]. We designed an ALU-GQ_RNA_ construct with this element in mind, since it is potentially a highly prevalent, biologically relevant ADAR1 target. With the ALU-GQ_RNA_, LLPS was more pronounced than with the U-loop-GQ, with all four ZBDs affected on a visually equivalent level.

**Figure 4.**
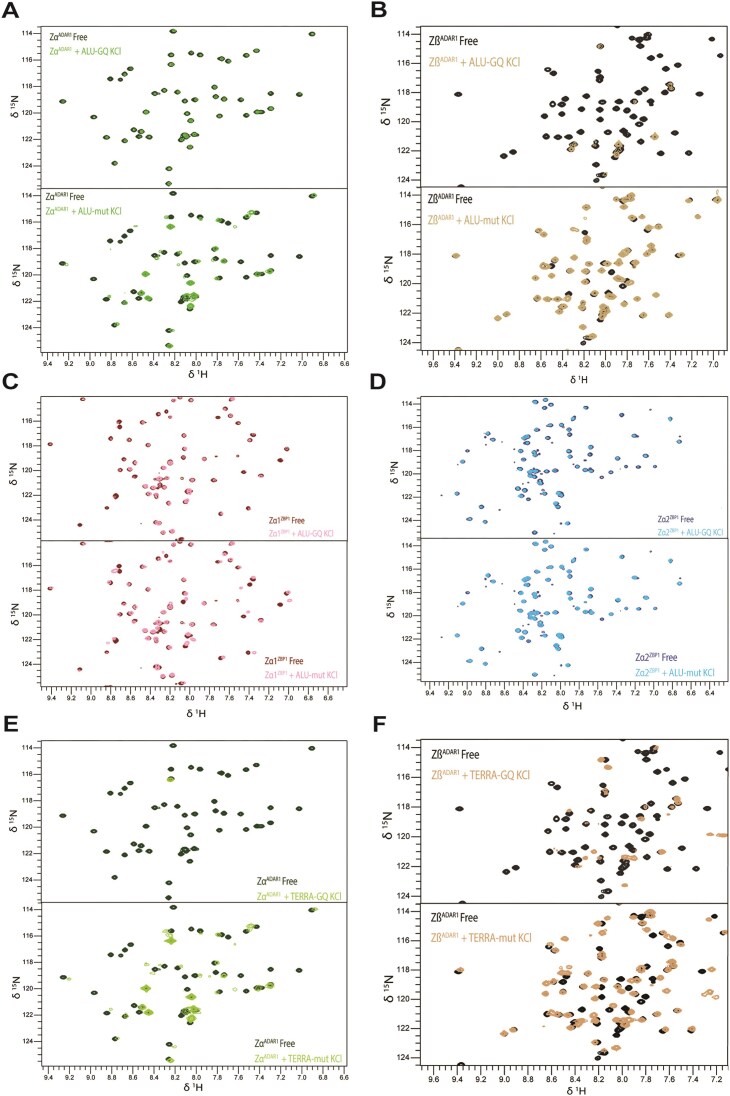
Comparison of Zα and Zβ domain binding to GQs or disrupted counterparts. Overlaid 2D HSQC spectra of free protein and ALU-GQ_RNA_ or ALU-mut_RNA_ in the presence of (**A**) Zα of ADAR1, (**B**) Zβ of ADAR1, (**C**) Zα1 of ZBP1, and (**D**) Zα2 of ZBP1. Full spectra are shown in Supplementary Fig. S5. Overlaid spectra of TERRA-GQ_RNA_ or TERRA-mut_RNA_ in the presence of (**E**) Zα of ADAR1 and (**F**) Zβ of ADAR1.

ADAR1 Zα showed no apparent peak shifts, but only peak height reductions (Fig. 4A and Supplementary Figs S5A and S6A). The peak height reductions were more intense than those observed for the U-loop-GQ_RNA_, and less than those of other ZBDs (Supplementary Fig. S6).

Zβ, unlike Zα, showed extensive peak disappearance with ALU-GQ_RNA_ (Fig. 4B and Supplementary Fig. S5B). The greater peak-height reduction and peak disappearance were more pronounced than that seen with U-loop-GQ and are the largest of any ZBD interaction with ALU-GQ_RNA_ (Supplementary Fig. S6B).

Zα1 displayed a similar spectrum with ALU-GQ_RNA_ as it did with U-loop-GQ_RNA_, showing some peak disappearance (Fig. 4C and Supplementary Fig. S5C). Curiously, despite seeing varying degrees of LLPS between the two constructs, the spectra remain nearly identical, and the average peak height reduction between these two experiments was nearly equivalent (Supplementary Figs S4C and S6C). These observations suggest that Zα1 binds similarly to both parallel strand GQ RNAs.

Zα2 demonstrated a different interaction with ALU-GQ_RNA_ than with U-loop-GQ_RNA_; despite similar levels of LLPS, we observed significant peak height reductions with ALU-GQ_RNA_ that were not seen with U-loop-GQ_RNA_ (Fig. 4D and Supplementary Figs S5D and S6D).

These results reveal that the interactions of the four ZBDs with the ALU-GQ_RNA_ are similar to those with U-loop-GQ_RNA_, but not equivalent. This could be due to sequence specificity or minor differences of the GQ structures resulting from the difference in the loop base. We also observed greater LLPS for Zβ with ALU-GQ_RNA_ than with U-loop-GQ_RNA_. Despite this outcome, both GQs produced similar large and specific changes to the Zβ spectra and peak height. Zα2 also showed a significant difference between the two GQs, with greater peak disappearance with ALU-GQ_RNA_ than with U-loop-GQ_RNA_ despite a similar level of LLPS. In contrast, Zα and Zα1 showed similar spectra for both U-loop-GQ_RNA_ and ALU-GQ_RNA_. Overall, for both RNA GQs, Zβ underwent the most significant spectral changes, followed by Zα2, Zα1, and lastly Zα.

##### Zα and Zβ domains interact with TERRA-GQ_RNA_

We also examined the binding interaction between ADAR1 subunits and the parallel-stranded TERRA-GQ_RNA_ construct that has UUA loops. As all ZBDs can interact with GQs, we focused on ADAR1, motivated by the previously published hypothesis proposing specific Zβ interactions with GQs. The TERRA-GQ_RNA_ construct is derived from the TERRA and forms a GQ under our experimental conditions (Supplementary Fig. S1A). Localization of both ADAR1 and ZBP1 to telomeric regions has been reported previously [34, 38]. However, the interactions have not been characterized at the molecular level. As a well-established GQ, the TERRA-GQ_RNA_ also serves as a positive control for our other constructs.

While undergoing LLPS, Zα still showed very extensive peak disappearance, indicative of tight binding to the TERRA-GQ_RNA_ (Fig. 4E and Supplementary Fig. S7A). These effects are in stark contrast to those previously seen for Zα with other GQs: the TTT-loop-GQ_RNA_ resulted primarily in shifts, while U-loop-GQ_RNA_ and ALU-GQ_RNA_ caused only peak height reductions. Thus, TERRA-GQ_RNA_ presents a unique binding interaction for Zα that may be tighter than with the other three examined GQs. Peak heights were almost entirely extinguished, rivaling the reductions seen for Zβ with previous RNA GQs (Supplementary Fig. S8A). It is worth noting that the two GQs resulting in visible HSQC changes for Zα (TTT-loop-GQ_DNA_ and TERRA-GQ_RNA_) are 25 and 24 nucleotides in length, whereas the shorter constructs (ALU-GQ_RNA_ and U-loop-GQ_RNA_ are 15 nucleotides each) show no changes to the spectra. This could be due to the inherently lower stability of the longer constructs, a preference for Zα to bind longer nucleic acids, or the effects of loop interactions. In contrast, binding of Zα to Z-NA requires a binding site of only 6 bp, without the marked LLPS formation observed with the GQ constructs [63].

Zβ also showed significant peak disappearance and shifts upon TERRA-GQ_RNA_ addition (Fig. 4F and Supplementary Fig. S7B). Similar to Zα, we observed considerable LLPS. The CSPs were large and, paired with extensive peak disappearance, indicate strong binding (Supplementary Fig. S8C, see later section 4.4). The appearance of peak shifts here indicates that Zβ is also interacting uniquely with the TERRA-GQ_RNA_ compared to the other three GQs, but the difference between these interactions is not yet known.

##### ADAR1 and ZBP1 ZBDs induce structural changes and phase separate upon G-quadruplex binding

**Figure 5.**
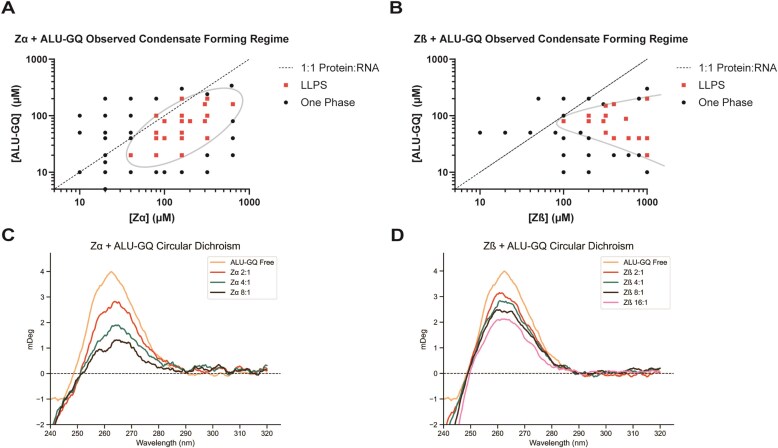
ADAR1 Zα and Zβ phase separate with ALU-GQ_RNA_ and have different GQ restructuring properties. Phase diagram plotting ZBD concentration versus ALU-GQ_RNA_ concentration, with observed phase boundary denoted for (**A**) Zα or (**B**) Zβ. CD measurements of constant ALU-GQ_RNA_ with increasing concentrations of (**C**) Zα or (**D**) Zβ.

As we have shown, all ZBDs form opaque material upon addition of both U-loop-GQ_RNA_, ALU-GQ_RNA_, and TERRA-GQ_RNA_. Thus, we set out to characterize the condensate in greater detail. To determine whether the gel-like material observed corresponded to a phase-separated state, we measured absorbance at OD_800_ [64] as a readout of sample turbidity. These measurements revealed that condensate formation was dependent on both protein and RNA concentration, requiring micromolar concentrations of both components (Fig. 5A and B).

Consistent with the known salt sensitivity of electrostatically driven phase separation [65–67], the condensates exhibited re-entrant behavior upon increasing ionic strength, with dissolution observed at 100 mM NaCl and further reduced at 200 mM NaCl. Also consistent, incubation at elevated temperature (42°C) similarly led to re-entrant dissolution [67]. Finally, variation of RNA and protein concentrations allowed us to establish a phase boundary separating the one- and two-phase regimes (Fig. 5A and B). Intriguingly, Zα appeared to exhibit some degree of time-dependent re-entry.

As ADAR1 Zα phase separated at lower concentrations of ALU-GQ_RNA_ than Zβ (Fig. 5A and B), we used CD to characterize the effect of protein binding to GQ RNA. We found that both Zα and Zβ caused a reduction in peak intensity of the GQ signal at 260 nM. This decrease is likely not due to LLPS effects, as the initial titration points are at low protein and nucleic acid concentrations (5 µM GQ, 10 µM ZBD) and should not be forming condensates.

At higher Zα to GQ ratios, the reductions in signal are likely due to a change in the structure of the GQ. Zα was a more potent ALU-GQ_RNA_ “melter,” showing a signal intensity reduction of four-fold at 8:1 Zα to GQ, whereas Zβ had only dropped two-fold by the 16:1 titration point (Fig. 5C and D). This difference in GQ melting could explain the observed differences in the phase diagrams; GQ multivalency is likely relatively low compared to ssRNA, and thus unwinding of GQ would increase its propensity to phase separate. Zβ, as a less effective GQ unwinder, has a corresponding phase boundary requiring higher RNA concentration.

This GQ structural change may be a consistent GQ binding effect at higher Zα to GQ ratios; when probing binding to TERRA-GQ_RNA_, we similarly see reductions in CD signal at 260 nM. However, in the case of this construct, Zβ induced a greater signal reduction than did Zα, with neither reaching the level seen with ALU-GQ_RNA_ (Supplementary Fig. S9A and B). In the case of Zβ with TERRA-GQ, signal reductions reached an apparent plateau at an 8:1 protein to nucleic acid ratio. Additionally, an apparent negative peak at 290 nM for Zα increased linearly with protein concentration, ruling out this peak as an effect of conversion to a new RNA structure (Supplementary Fig. S9C). Overall, these results are consistent with a model of binding induced structural change that still retains significant GQ structure and varies with the loop composition.

Our experiments do not reveal what GQ structural rearrangement is occurring. 1D NMR visualization of bound RNA is often occluded by large complex formation, preventing characterization via imino peaks. It is possible that the ZBDs are fully unwinding some GQs (especially in the case of Zα with ALU-GQ_RNA_), or they could be rearranging the structure into a more open form that retains significant GQ character, hence the remaining peak intensity via CD. In either case, GQ-mediated ZBD phase separation is a compelling finding that may have biological implications. For example, the unwinding of GQs in stress granules may promote cross-hybridization between mRNAs as they mature into irreversible aggregates [68].

#### ZBPs interact with a broad range of nucleic acid folds

Zα domains are known to bind to Z-NA, as well as A-RNA and B-DNA, albeit with weaker affinity [69]. In contrast, Zβ has not been shown to strongly bind any nucleic acids to date. While we have shown that all ADAR1 and ZBP1 ZBDs bind to GQs, their interactions appear to depend on GQ architecture. Therefore, we sought to investigate whether these domains bind nucleic acid motifs other than GQs (and A-, B-, and Z-form double-stranded nucleic acids in the case of Zα domains) to determine whether ZBDs bind GQs with greater specificity.

##### ADAR1 and ZBP1 ZBDs interact with disrupted G-quadruplexes

To assess if ZBDs bind GQs with structure‐specific recognition, we generated mutant ALU and TERRA constructs that interrupt the formation of G-tetrads and therefore prevent GQ formation (Supplementary Fig. S1A). Mutation of each GGG repeat to CCG (TERRA-mut_RNA_, ALU-mut_RNA_) successfully disrupted the GQ structure (Supplementary Fig. S1A). Based on melting temperatures and CD spectra, we can infer that these mutated sequences are forming some higher order structure (Supplementary Fig. S1A and C). However, lack of peaks in the imino region of 1D ^1^H NMR spectra indicates that they do not form duplexes, but may adopt transient and unstable structures, thereby retaining significant single-stranded character, or adopt conformations similar to those found in tetraloops stabilized by base-stacking interactions rather than by hydrogen bonding [70].

Zα exhibited extensive peak shifts with both mutant constructs, in contrast to the previous results, which showed only peak disappearance. TERRA-mut_RNA_ induced larger shifts than ALU-mut_RNA_ (Fig. 4A and E; Supplementary Fig. S8E and S11A). Zα did exhibit some LLPS with the ALU-mut_RNA_, albeit to a lesser extent than seen with U-loop-GQ_RNA_ or ALU-GQ_RNA_. Notably, we observed greater peak-height reduction here than with any previous construct except TERRA-GQ_RNA_ (Supplementary Fig. S10A). TERRA-mut_RNA_ binding yielded even greater levels of peak-height reduction than with ALU-mut_RNA_, second only to TERRA-GQ_RNA_ (Supplementary Fig. S8A and B). Therefore, Zα is likely binding TERRA-mut_RNA_ tightly, as peak disappearance appears to be a result of the binding interaction rather than phase separation.

Zβ also bound both constructs, which primarily resulted in peak shifts (Fig. 4B and F; Supplementary Figs S8F and S11B). Intriguingly, titrating ALU-mut_RNA_ into Zβ resulted in no LLPS, and we observed less peak disappearance compared to our other GQs (Fig. 4B and Supplementary Fig. S10B). TERRA-mut_RNA_ caused larger shifts than those seen for ALU-mut_RNA_. However, TERRA-GQ_RNA_ addition still resulted in a markedly greater reduction of peak intensity than TERRA-mut_RNA_ and is likely binding tighter (Supplementary Fig. S8C and D). Taken together, Zα and Zβ bind to both GQ and mutant RNA constructs, with both ZBDs binding with different modes/dynamics to these various structures.

Zα1 behaved similarly to Zβ when bound to ALU-mut_RNA_; minimal precipitation was observed. While ALU-GQ_RNA_ induced significant peak disappearance with Zα1, ALU-mut_RNA_ primarily yielded peak shifts and caused less peak-height reduction than any GQ tested with Zα1 (Fig. 4C and Supplementary Figs S10C and S11C).

Zα2 displayed a distinct interaction with ALU-mut_RNA_. We observed substantial phase separation upon addition of the ALU-mut_RNA_, but the resulting HSQC spectra showed a significant lack of reduction to peak intensity with minimal chemical shift changes (Fig. 4D and Supplementary Figs S10D and S11D).

These results indicate that ZBDs are capable of binding nucleic acids beyond canonical duplex DNA/RNA, Z-form motifs, and GQs. However, the interactions with ALU and TERRA mutant sequences differ from those with their GQ counterparts. With Zβ, Zα1, and Zα2 the interactions with mutant sequences appear weaker than those observed with wild-type GQs, whereas for Zα, the spectral changes are notably more distinct than those seen with most GQs.

##### Buffer ion affects ADAR1 Zβ but not Zα binding to ALU and U-loop-GQ

While our previous measurements were conducted in GQ promoting K^+^ buffer, the following experiments were performed in Li^+^ buffer to assess whether interactions reflect structural or sequence specificity. As shown, the buffer ion markedly influences the stability of GQ, with K^+^ having the strongest stabilizing effect and Li^+^ the weakest (Supplementary Figs S1C, E, and S2). We hypothesized that modulating the predominant ion in buffer would allow further examination of the specificity of ZBD-GQ interactions. Even though a GQ in Li^+^ is less stable, we were still able to observe LLPS in LiCl buffer.

As Zα exhibited no significant changes to its spectrum when interacting with ALU-GQ_RNA_, it is unsurprising that the spectral peak positions and intensities were similar with KCl and LiCl buffers (Supplementary Figs S6A, S12B, and S13B). These observations suggest that Zα does not exhibit a strong preference for the stability or the fold state of ALU-GQ_RNA_. We saw similar interactions with U-loop-GQ_RNA_, where neither buffer condition induced large peak disappearance or shifts (Supplementary Figs S12A and S13A). In fact, we observed slight decreases in the peak heights in the Li^+^ over the K^+^ sample (Supplementary Figs S4A and S13A), although these variations were minor.

Zβ exhibited a strong response to buffer ion. While we saw extensive peak disappearance with ALU-GQ_RNA_ and U-loop-GQ_RNA_ in KCl, the vast majority remained visible in Li^+^ (Supplementary Fig. S12C and D). These changes, together with a large relative increase in peak height (Supplementary Figs S4B, S6B, and S13C and D), suggest a strong difference in binding between buffer conditions. Generally, the effects were slightly less pronounced for U-loop-GQ_RNA_ than for ALU-GQ_RNA_. Overall, these data indicate that Zβ is sensitive to buffer ion when interacting with GQs, implying that GQ stability, and potentially its structure, modulates binding.

##### ADAR1 Zβ binds preferentially to an intermolecular G-quadruplex compared to its individual strands

To further test structural preference over sequence preference, we generated two 6-mer constructs derived from the TERRA-GQ_RNA_ and TERRA-mut_RNA_ sequences (UUAGGG, UUACCG). NMR confirms that both UUAGGG and UUACCG are predominantly single-stranded at low concentrations, while at higher concentrations, UUAGGG forms an intermolecular GQ (ssUUAGGG; 30 uM versus GQ-UUAGGG; 100 uM) (Supplementary Fig. S14A and B). The ability to control formation of GQ in solution by adjusting concentration would allow us to directly examine the structural specificity of the ZBDs by eliminating any variables regarding sequence or buffer condition. Additionally, neither Zα nor Zβ underwent phase separation upon addition at either low or high concentration; this could indicate LLPS being a NA length dependent effect or even a GQ structural effect.

Zα showed mild interaction with UUACCG which increased with concentration, confirming Zα’s ability to bind to ssRNA (Supplementary Figs S14C, S15A, S16A and B, and S17). UUAGGG at low concentrations showed a similar degree of CSPs as with UUACCG at high concentration; this may suggest a slight guanine base preference (Supplementary Figs S14D, S15B, and S16C). At high concentration of UUAGGG, which has a greater population of GQ and somewhat reduced peak heights compared to the other interactions (Supplementary Fig. S16A–D), we saw a similar level of CSPs as with the predominantly ssUUAGGG sample (Supplementary Figs S14D, S15B, S16D, and S17). The similarity in binding between these two populations may suggest that the binding mode is similar between these two structural states. Overall, Zα is capable of binding to ssRNA and appears to do so in a similar manner as to GQ binding.

Zβ showed weak binding with UUACCG at both low and high concentrations and very weak binding with UUAGGG at 30 µM (Supplementary Figs S14E, S15C, and S16E–G). This suggests that Zβ does not strongly bind ssRNA and has no guanine base preference. However, for the higher GQ population sample, we saw a large increase in CSP and reduced peak heights (Supplementary Figs S14F, S15C, and S16E–H). These results show that Zβ has a strict GQ structural preference rather than a guanine base preference.

In agreement with these observations, previous results indicated that both Zα and Zβ were capable of interacting with CpG-repeat ssRNA at very low concentrations [71]. Altogether, these results confirm that while both Zα and Zβ are potent GQ binders, Zβ may have a stricter structural preference rather than sequence specificity.

##### ADAR1 Zα and Zβ have nM to µM binding affinities with G-quadruplexes

To further probe the nature of GQ binding, we sought to measure binding affinities for our various RNA and DNA constructs. As NMR results indicated differential binding between GQs, affinity measurements would both quantify the strength of ZBD–GQ interactions, while also illuminating key differences in interactions between GQ structures.

Given the trends of peak position changes and disappearance observed in the NMR spectra of the ZBDs upon addition of various nucleic acid constructs, the typical *K*_d_ appears to fall in the nanomolar to low micromolar range. However, because peak disappearance is often the primary effect of binding, a more refined quantification by NMR titration experiments is challenging for many interactions studied here. When feasible, we performed microscopic *K*_d_ analysis using NMR titration to measure binding affinity. In addition, we also used orthogonal methods to quantify these interactions under different experimental conditions, conducting both select ITC determinations and MST.

**Figure 6.**
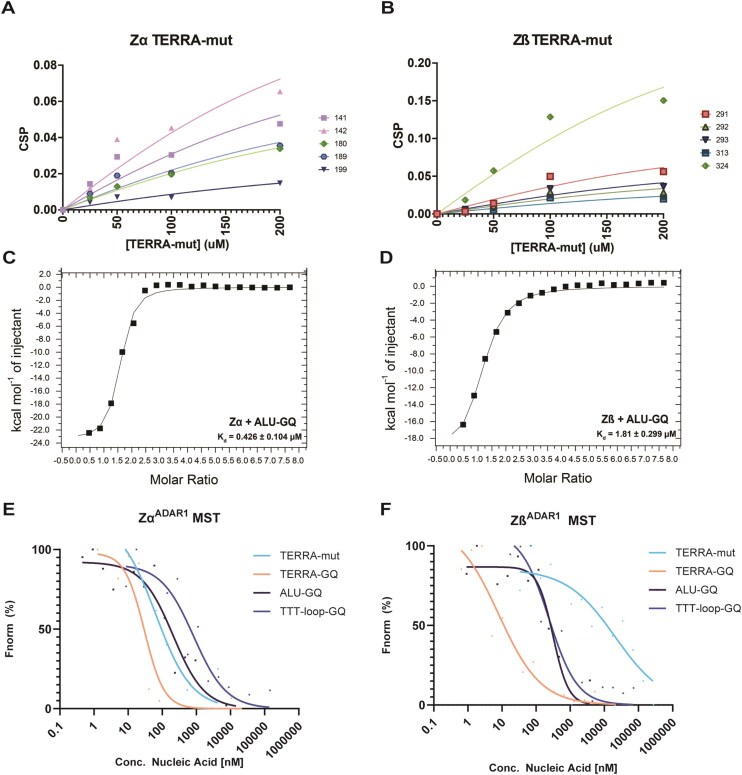
Affinity analysis of the interaction of ADAR1 Zα and Zβ domains with NA constructs. Residue-specific NMR CSP-based binding affinity curves for (**A**) Zα and (**B**) Zβ binding to TERRA-mut_RNA_. Highlighted residues are shown in the legend. ITC curves of (**C**) Zα and (**D**) Zβ binding to ALU-GQ_RNA_. MST curve fits at 1.5 s. for (**E**) Zα and (**F**) Zβ.

We initially extracted affinities for both ADAR1 ZBDs with TERRA-mut_RNA_ using NMR. These are microscopic affinities that provide residue-by-residue values. *K*_d_ values were determined by fitting peak position changes, that is, CSP as a function of ligand concentration (Supplementary Fig. S22A and B). We obtained overall affinities of ~108 µM for Zα and 42 µM for Zβ (Fig. 6A and B). While Zα retained many of its peaks, it underwent significant peak disappearance for most critical residues. These values are likely representative of a global effect of binding to TERRA-mut_RNA_ for which we do not have direct data on Zα’s binding site. Given the magnitude of peak disappearance with TERRA-mut, the true affinity is likely much stronger.

We then used ITC to measure the affinity between both ADAR1 ZBDs with ALU-GQ_RNA_ (Table 2), given the potential role this fold plays in targeting ADAR to ALU repeat editing substrates. We obtained low-micromolar affinity for both Zα and Zβ when binding to this GQ. Zα bound with a *K*_d_ of 0.43 ± 0.10 µM. In contrast, Zβ exhibited weaker binding, with a *K*_d_ value of 1.81 ± 0.30 µM. (Fig. 6C and D; Supplementary Fig. S20). The entropy, enthalpy, and *N* values from these experiments are also shown in Table 2. ITC conditions are near the LLPS boundary but are still not ideal. We therefore conducted affinity measurements using MST, where we could use conditions well below the threshold for LLPS to occur.

We used MST to interrogate RNA/DNA GQ as well as Mut_RNA_ interactions with both Zα and Zβ of ADAR1 (Fig. 6E and F; Table 3). All MST binding fits are summarized in Table 3. Due to unusual fluorescence changes at high nucleic acid concentration at late timepoints in the MST spectra, we used the 1.5 s time point fit post-IR laser shining for all measurements to extract affinities. A reversal in thermophoretic response was observed at higher RNA concentrations, which may reflect additional concentration-dependent interactions, potentially arising from transient multivalent contacts. Notably, this behavior was absent at lower concentrations, where the signal was consistent with canonical binding of Zα and Zβ to the RNA constructs.

**Table 3.** MST *K*_d_ values

Domain	ALU-GQ_RNA_	TERRA-GQ_RNA_	TTT-loop-GQ_RNA_	TERRA-mut_RNA_	ALU-mut_RNA_
ADAR1 Zα	115.5 ± 69.3 nM	19.3 ± 13.7 nM	462.5 ± 67.5 nM	35.9 ± 8.9 nM	Cannot fit
ADAR1 Zβ	336.4 ± 181.0 nM	4.2 ± 2.2 nM	210.07 ± 49.1 nM	22 470 ± 14 600 nM	Cannot fit

**Table 4.** Type of interaction observed in HSQC NMR spectra

Domain	U-loop-GQ_RNA_	ALU-GQ_RNA_	ALU-mut_RNA_	TERRA-GQ_RNA_	TERRA-mut_RNA_	TTT-loop-GQ_DNA_
ADAR1 Zα	PI *	PI **	CS **	PI ***	CS ***	CS, PI **
ADAR1 Zβ	PI *	PI **	CS ^x^	CS, PI ***	CS ^x^	CS, PI **
ZBP1 Zα1	PI *	PI **	CS ^x^	-	-	CS, PI *
ZBP1 Zα2	PI *	PI **	PI ^x^	-	-	CS, PI **

CS = chemical shift changes, PI = peak intensity changes. *, **, *** indicate increasingly stronger nM range binding, x indicates μM range binding.

With a *K*_d_ value of 19.3 ± 13.7 nM, TERRA-GQ_RNA_ showed the strongest measured interaction with Zα, in agreement with the NMR-based results. Interestingly, Zα bound TERRA-mut_RNA_ with a comparable affinity (35.9 ± 8.9 nM), suggesting that the unknown structure formed by this element, along with unpaired bases also interacts with Zα. The interaction of Zα with ALU-GQ RNA is weaker (*K*_d_ = 115.5 ± 69.3 nM). TTT-loop-GQ_DNA_ had an even weaker interaction (*K*_d_ = 462.5 ± 67.5 nM). When measurements were recorded for the interaction of Zα with ALU-mut_RNA_, no fit was found to the data (Supplementary Fig. S18A). However, we were able to use initial fluorescence to estimate the affinity of Zα for this construct. Binding always resulted in ligand-induced fluorescence changes (not due to ligand autofluorescence or GQ binding to dye) (Supplementary Fig. S19A). The analysis indicated that Zα initially bound ALU-mut_RNA_ to a similar extent as ALU-GQ_RNA_ (Supplementary Fig. S19B), recapitulating the trends observed in the MST results. Finally, Zα had the weakest interaction with TTT-loop_DNA_, binding ∼one order of magnitude weaker than the other constructs. Additionally, we conducted a competition assay between TERRA-GQ_RNA_ and (CpG)_12_ RNA, a dsRNA construct of the same length as TERRA-GQ_RNA_. We have previously shown that Zα of ADAR1 binds CpG repeats with affinities of a few 100 nM [69] and efficiently converts (CpG)_12_ to Z-form. [71] Initial fluorescence fits showed that TERRA-GQ_RNA_ still bound Zα with nM affinity in the presence of (CpG)_12_ amounts saturating Zα; while we obtained a slight decrease in affinity, the affinity was not statistically different from the non-competition TERRA-GQ_RNA_ binding affinity (*P* = .0635). This indicates that TERRA-GQ_RNA_ is able to outcompete dsRNA binding to Zα (Supplementary Fig. S21A).

In agreement with our NMR titration measurements, Zβ bound TERRA-mut_RNA_ only weakly in the mid µM range. With ALU-mut_RNA_, as was the case for Zα, we were not able to fit a curve to the data (Supplementary Fig. S18B). ALU-GQ_RNA_ and TTT-loop_DNA_ bound in the high nM range (*K*_d_ values of 336.4 ± 181.0 nM and 210.1 ± 49.1 nM), bordering on two orders of magnitude tighter than binding to TERRA-mut_RNA_. Finally, Zβ bound TERRA-GQ_RNA_ with an unexpectedly tight affinity in the low nM range with a *K*_d_ of 4.2 ± 2.2 nM (Fig. 6F). This interaction is more than an order of magnitude tighter than with either of the other two measured GQs (and more than three orders greater than with TERRA-mut_RNA_). The binding is tighter than any of the measured affinities for Zα. However, this affinity measurement for TERRA-GQ_RNA_ was obtained within the ligand-depletion range and may not be exact. We validated these results using initial fluorescence, yielding similar trends for each GQ examined (Supplementary Fig. S19C). Neither ALU-mut_RNA_ or TERRA-mut_RNA_ yielded fittable initial fluorescence, further demonstrating weak binding of Zβ to non-GQ structures (Supplementary Fig. S19D). Thus, Zβ exhibits a wide range of specificity; weak binding with TERRA and ALU mutants, ‘medium’ binding with some GQs (ALU, TTT-loop, and likely U-loop), and very tight binding with TERRA-GQ_RNA_. Finally, we conducted a similar competition assay as done for Zα with Zβ between TERRA-GQ_RNA_ and (CpG)_12_, and found no significant difference between the two initial fluorescence fitting curves (*P* = .3574), further showing Zβ’s strong preference for GQs (Supplementary Fig. S21B).

Because of unusual fluorescence behavior, some errors in the *K*_d_ fits were relatively large. Nevertheless, they serve well as comparative tools for the binding strengths of nucleic acid constructs to ZBDs. Given the similar trends obtained from NMR, ITC, and MST measurements with vastly different protein concentrations, these measurements are likely close to the “true” binding affinities. We also note that the MST measurements were recorded at low nM protein concentrations and thus should be independent of any phase separation properties. Overall, the results reflect tight interactions of both ADAR1 ZBDs, with Zβ exhibiting unique GQ specificity.

##### NMR confirms the nucleic acid binding preference of ADAR1 Zα and Zβ

Despite extensive peak disappearance in the HSQC spectra of the ADAR1 ZBDs in complex with many constructs (Zα with TERRA-GQ_RNA_, Zβ with ALU-GQ_RNA_, U-loop-GQ_RNA_, TERRA-GQ_RNA_), other constructs instead show CSPs with varying degrees of peak shifts. For Zα, we obtained measurable CSPs for both mutant sequences and the TTT-loop-GQ_DNA_, as well as minor but consistent CSPs for U-loop-GQ_RNA_. Notably, certain residues consistently shifted in the same direction upon the addition of nucleic acid (Supplementary Fig. S23A), allowing us to confirm the trends from MST using shift distance as a proxy for binding strength.

When comparing NA constructs in interaction with Zα, a trend emerged: the two non-GQ constructs (TERRA-mut_RNA_ and ALU-mut_RNA_) consistently exhibited the greatest magnitude of CSPs, whereas the two GQ constructs with visible CSPs (TTT-loop-GQ_DNA_ and U-loop-GQ_RNA_) exhibited smaller changes (Supplementary Figs S23A and B). These trends are consistent with our affinity measurements, in which TERRA-mut_RNA_ yields a tighter affinity than these GQs, while Zα interacts weakest with the TTT-loop-GQ_DNA_. Additionally, several peaks shift in a different direction with TTT-loop-GQ_DNA_ than any other construct, which could be a result of differential interactions with RNA and DNA, or due to differences in the structure of parallel and anti-parallel GQs.

Some GQ constructs retained sufficient peak intensity after the addition of Zβ to analyze CSPs. We compared TERRA-mut_RNA_, ALU-mut_RNA_, TTT-loop-GQ_DNA_, and TERRA-GQ_RNA_. Once again, a consistent trend emerged here: TERRA-GQ_RNA_ peaks always shifted further than or equal to the other constructs, in accordance with its tight affinity measured via MST. These results further confirm both the affinity measurements as well as the 6-mer HSQC spectra, indicating that Zβ, but not Zα, has GQ structural specificity, as well as tighter binding of Zβ to specific GQs than Zα.

#### ADAR1 domains bind to G-quadruplexes primarily via α-helices

Having observed that specific residues in both Zα and Zβ exhibit consistent CSPs upon interaction with GQs, we can extract information regarding the binding interface with GQ nucleic acids. In principle, similar information could be obtained from peak-height intensity plots. However, for constructs that show no peak shifts and only peak disappearance, it is not possible to distinguish residues involved in direct interaction from those peaks that disappear due to slowed overall tumbling. Therefore, we analyzed only spectra showing CSPs to investigate the binding interface. Significant CSP values were defined as (CSP − Mean)/STD > 1.

**Figure 7.**
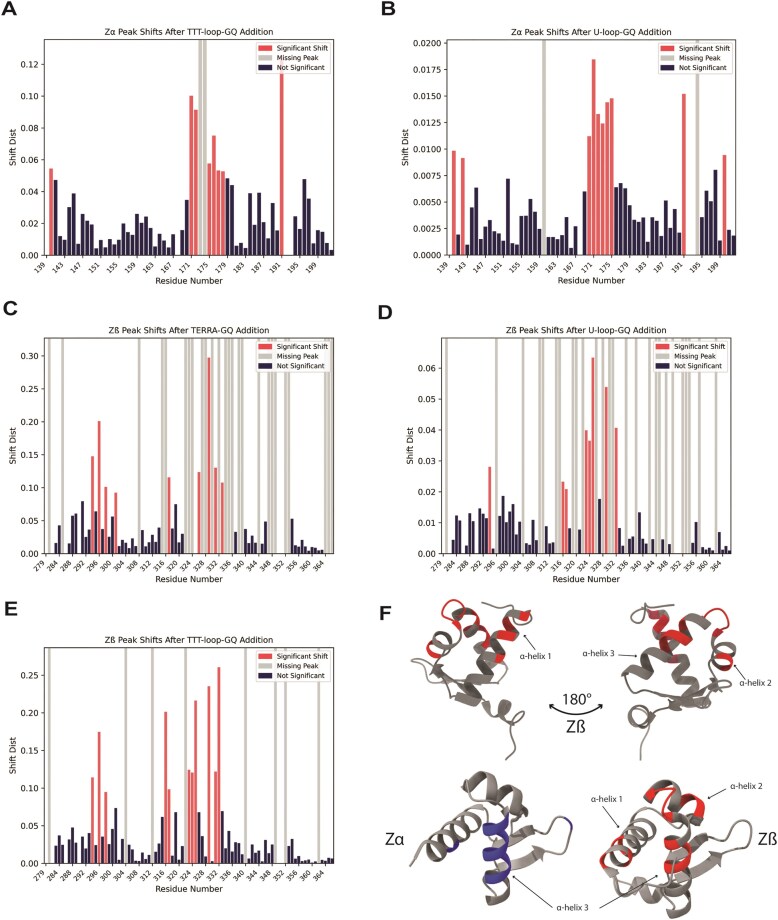
Sites of interaction of ADAR1 Zα and Zβ upon GQ binding. CSPs obtained from HSQC spectra of Zα upon addition of (**A**) TTT-loop-GQ_DNA_ and (**B**) U-loop-GQ_RNA_ in KCl. CSPs obtained from HSQC spectra of Zβ upon addition of (**C**) TERRA-GQ_RNA_, (**D**) U-loop-GQ_RNA_ in KCl, and (**E**) TTT-loop-GQ_DNA_. Red, blue, and gray bars represent significant CSPs, non-significant CSPs, and gray bars missing/disappeared peaks. (**F**) Structure of Zβ with residues significantly affected upon addition of GQs highlighted in red (top), and comparison of aligned Zα and Zβ, with significant residues highlighted in blue and red, respectively (bottom).

We compared our Zα binding site results with those presented in a previous study that demonstrated interactions with a DNA GQ [37]. In that study, Zα engaged the GQ primarily through α-helices 2 and 3 and the β-wing, affecting residues Ala158, His159, Lys169, Lys170, Glu171, Asp173, Arg174, Val175, Lys176, Lys182, Ala189, and Thr191. These residues substantially overlap with those that mediate Z-NA binding, for which Tyr177 and Trp195 are also critical. Based on the CSPs of the TTT-loop-GQ_DNA_ and U-loop-GQ_RNA_ (Fig. 7A and B), we found that the primary residues interacting with our GQs are Glu140, Lys170, Glu171, Ile172, Asp173, Arg174, Val175, Leu176, Tyr177, Ser178, and Thr191. This set is highly similar to that previously reported for GQ binding and overlaps extensively with the Z-NA interaction interface. These residues similarly map to α-helix 3 and the β-wing.

CSP analysis of Zβ interactions with TERRA-GQ_RNA_, U-loop-GQ_RNA_, and TTT-loop-GQ_DNA_ indicates primary affected residues across these three constructs being: Leu294, Met296, Glu298, Leu316, Asn317, Gly323, Leu324, Thr325, Asp329, Asn331, and Ala332, which are affected in binding to at least two of the three GQs. We also see the consistent disappearance of residue Ile352 in the β-wing. Residues Glu301, Lys326, and Val333 show significant CSPs only in TERRA-GQ_RNA_. These residues do shift in both other constructs, but not to a significant level. Finally, Arg328, which was hypothesized to play a significant role in GQ interaction [49], disappeared completely in all GQ binding spectra, but exhibited large shift distances in both mutant sequences. Taken together, these data indicate that the Zβ GQ binding interface is formed primarily by α-helices 1 and 3, with contributions from portions of α-helix 2 and the intervening loop between α-helices 2 and 3.

We also examined the binding of a giant virus Zα domain which shares significant sequence similarity with both ADAR1 Zα and Zβ, particularly in the α-helix 3 region (Supplementary Fig. S24B) [55]. We found that it bound to ALU-GQ_RNA_ with a somewhat weaker affinity (*K*_d_ ∼ 1 μM) than ADAR1 Zα or Zβ, further confirming that these conserved residues are key interactors in GQ binding (Supplementary Fig. S24A).

Intriguingly, a comparison of the Z-NA binding interface of Zα to the same region of Zβ implicates several critical residues in Zα that diverge in Zβ resulting in an inability to bind Z-NA, two of which being Arg174/Ala332, and Tyr177/Ile335^20^. The most critical residue implicated in Zβ’s lack of Z-NA binding is Tyr177/Ile335; Ile335 peaks have disappeared in Zβ binding to several GQs, and present slight changes in other GQs and the mutant sequences. Ala332 is also one of the most strongly shifting residues across all constructs, suggesting that it is critical for recognition of both GQs and ssRNAs. Despite Zβ sharing a common binding interface for both NA folds, α-helix 1 may contribute additional GQ specificity, as this region is more perturbed by GQs than by mutants (Fig. 7C–E and Supplementary Figs S8F and S10B) Overall, both ZBDs utilize similar regions within α-helix 3, but Zβ additionally incorporates α-helices 1 and 2 into its GQ binding surface (Fig. 7F).

### Discussion

**Figure 8.**
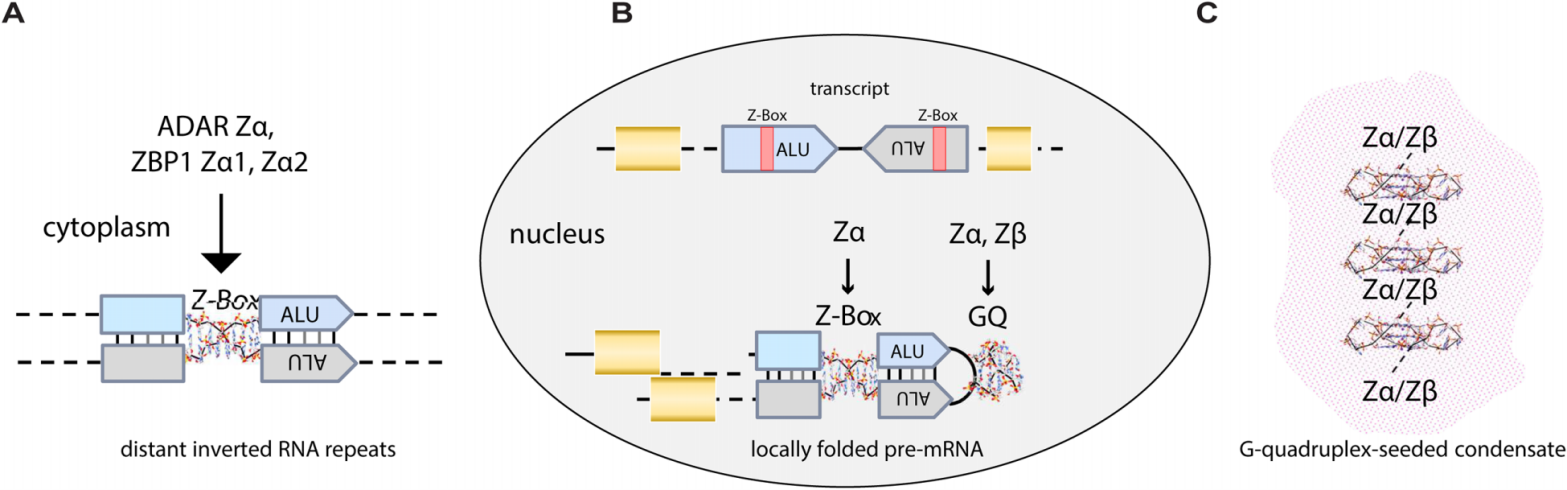
The physiological ligands of ZBPs involved in host defense against retroviral elements. (**A**) Zα domains of ADAR1 and ZBP1 can engage Z-prone regions in dsRNAs found in the cytoplasm, including the Z-Box (a Z-NA prone region) in IR-ALU elements. (**B**) Proposed model for ZBD engagement of alternative nucleic acid folds in the nucleus. Zβ binding to GQs in nascent transcripts could potentially localize ADAR1 to edit these RNAs and modulate splicing. GQs may also form in the loop separating the two arms of the IR-ALU. ADAR1’s Zα domain and ZBP1’s Zα1 and Zα2 domains can also bind GQs, but with lower specificity and through a different binding mode compared to Zβ. (**C**) Docking of Zα or Zβ domains to GQ arrays, such as those produced by telomeric DNA or RNA, can lead to condensate formation and activation of downstream pathways such as autophagy and cell death.

While it is well established that Zα domains of ADAR1 and ZBP1 bind nucleic acids with propensity to form Z-DNA and Z-RNA (Fig. 8A), it has also been shown by NMR that Zα of ADAR1 binds an oncogenic c-Myc DNA GQ [37]. However, no binding partner of the structurally homologous Zβ domain of ADAR1 has been identified to date, and the basis for its evolutionary conservation remains unresolved. Based on AlphaFold [51] and molecular dynamics simulations, we recently suggested that Zβ of ADAR1 targets substrates by recognizing GQs [49]. Here, we significantly extended the range of experimentally confirmed Zα–GQ interactions by demonstrating that the Zα domains of both ADAR1 and ZBP1 bind to both DNA and several RNA GQs.

We have also provided the first evidence of strong Zβ interaction with any substrate by showing direct, *in vitro* experimental evidence for Zβ binding to select GQ RNAs and DNAs. We find that not only is Zβ capable of binding to GQs with a tighter affinity than seen with any other substrate, but we also show that it exhibits strict GQ specificity rather than guanine base preference.

Finally, we demonstrated that all ZBDs are capable of binding to ssRNA/non-canonical RNA structure, showing varying degrees of interaction strength, Zα and Zβ domains interacted with mutant GQ sequences. This further expands the range of confirmed binding targets of these domains. Interestingly, these interactions may also be relevant to the role of loop composition in the affinity of Zα for GQ. An AlphaFold model of a “spare tire” GQ, consisting of an extra UUAGG repeat, reveals that the additional loop residues may enhance the stability of the interaction [72] (Supplementary Fig. S25). Similar effects may account for the differences in affinity observed for the GQ constructs studied here, and to the higher-order structures formed by our non-GQ forming controls (Supplementary Fig. S1A and C). To demonstrate trends between constructs, we categorized our HSQC observations. Although LLPS prevents the direct correlation of NMR spectral changes to binding affinity, we can use peak height reduction to assess the strength of interactions. The trends we observed are visualized in Table 4. Each ZBD interacted with all NA constructs tested in this study. However, the interaction fingerprints differ across constructs; Zβ, binds weakly to non-GQ RNA but very strongly to GQs. Zα of ADAR1 binds tightly with GQs, but also binds to another fold formed by our mutant constructs.

We further demonstrated that ZBDs interact with both parallel and antiparallel structures. The antiparallel TTT-loop-GQ_DNA_ exhibited extensive peak shifting, with a notable lack of peak disappearance in contrast to our other GQs. Interactions unique to the TTT-loop-GQ, such as certain Zα peaks shifting in different directions, could potentially be attributable to it being the only DNA GQ and the only antiparallel GQ examined in this study. Additionally, Zα exhibited a preference for both RNA-GQ and mutant RNA over the TTT-loop-GQ DNA, whereas Zβ had a preference for the DNA GQ over the mutRNA, binding comparably to our RNA-GQs. These results indicate that ZBDs may recognize both DNA/RNA and parallel/antiparallel GQs through different binding modes.

Additionally, we examined the binding interface used by ADAR1 ZBDs to recognize GQ. Overall, our data agree with the previously proposed regions of interaction [27]. Zα engages GQ using essentially the same surface that it uses to bind Z-NA, namely α-helix 3 and the β-wing. In contrast, Zβ interacts with GQs primarily through α-helices 1 and 3 and the loop between α-helices 2 and 3 (Fig. 7F). These data provide preliminary evidence that residues within the α-helix 1 region of Zβ contribute to preferential GQ recognition, as Zα does not utilize α-helix 1 in its binding surface. Furthermore, Zβ consistently shows significant CSPs in non-polar, aliphatic residues such as isoleucine and alanine; these residues are not typically involved in nucleic acid-binding interactions with B-DNA or A-RNA. However, our observations are consistent with those for the yeast RAP1 transcription factor, which has been crystallized with both B-DNA containing its cognate binding site and a parallel-strand GQ [73, 74]. The same RAP1 helix interacts with both conformations, but through different faces. The charged face binds to B-DNA and the hydrophobic face to the hydrophobic end caps of the GQ in a similar manner by which small molecules have been shown to bind GQs [41]. Likewise, we propose that non-polar residues in the ZBD α-helices are hydrophobically interacting with the top or bottom face of GQs, with specific charged residues (such as Arg328) contributing to the electrostatic interactions.

Two Zβ residues likely involved in binding to GQs, Ala332 and Ile335, are the positional equivalents of Arg174 and Tyr177 in Zα. Loss of these specific side chains explains Zβ’s inability to bind Z-NA. Their strong involvement in GQ binding suggests that the evolution of Zβ may have favored GQ specificity at the expense of Z-NA recognition. The precise functional role of these residues remains unknown, and further structural work is needed to characterize the binding interface better. It would also be interesting to further investigate these modes of binding to determine the mechanism by which ZBD LLPS occurs.

The findings support a model we previously proposed [49] (Fig. 8B), in which Zβ binds GQ formed co-transcriptionally by ALU repeats and other repeats with a GQ motif. The model further predicts that these GQs promote the localization of ADAR1p110 and the editing of dsRNAs formed by ALU inverted repeats, which might otherwise result in detrimental outcomes due to mis-splicing of the pre-mRNA. The recognition of GQs by ADAR1p110 may also promote the resolution of telomeric R-loops [38]. Of note, similar to A-to-I edits, GQs are enriched in human RNA intron splice sites [75]. The role of GQ recognition by ADAR1 in these processes may extend beyond dsRNA editing, as catalytically dead ADAR1 can still alter splicing patterns in mice [53]. Similar to Z-NA recognition by Zα, this mechanism may defend the host against retroelements and exploit the structures they form for other purposes. In the case of GQ, Zβ potentially helps regulate alternative splicing, miRNA processing, and exon recoding. An assessment of A-to-I edits suggested that the associated GQs were usually <175 bases from the editing site [49]. This distance is much less than the 7 kb separations possible for ALU inverted repeats that pair to form editing substrates [28].

Our results also provide new insights into the various roles played by Zα. We present the first experimental evidence for direct binding of ADAR1 Zα to TERRA, extending beyond the previously reported co-localization of ZBP1 to TERRA [34]. Indeed, both the Zα and Zβ domains of ADAR1 show a preference for the TERRA-GQ_RNA_ over other GQs. To confirm specificity for GQ, we examined Zα and Zβ binding to the TERRA-mut_RNA_, in which tetrad formation was disrupted by replacing guanine with cytosine. The affinity for these substrates was several orders of magnitude lower for Zβ than for the GQ fold. The unexpectedly high affinity of Zβ for TERRA-GQ_RNA_, relative to other GQ substrates, is a notable finding with potentially broad biological implications. Given previous results demonstrating localization of these proteins to the TERRA region, it becomes evident that the TERRA-GQ may be of specific importance to the biological function of ADAR1 and ZBP1. Experiments confirming ADAR1 ZBD binding to TERRA-GQ *in vivo* are necessary to further elucidate the role of GQ binding, and in particular the role that Zβ may play in biological systems. It is also interesting to speculate on the function that Zβ executes for ADAR1 in competition with ZBP1 for GQs, as both proteins were shown to target the TERRA region. As we have demonstrated that Zβ has a lesser ability to affect the structure of a GQ, it may play a protective role.

Our findings highlight the distinct modes by which Zα and Zβ target ADAR1 to different sites in the cell, enabling a multi-layered host defense against retroelements and pathogens. GQs can originate from a ssRNA produced during active transcription to localize ADAR1 to IR-ALUs that are then edited. Alternatively, ssRNAs that form GQs may localize ADAR1 to modulate splicing in an editing-independent manner [53]. These events affect the processing of pre-mRNAs and occur in the nucleus. In the cytoplasm, the formation of Z-RNAs is promoted by helicases and by torsional strains that can be found in stress granules. ADAR1p150 has been shown to localize to stress granules via its Zα domain to negatively regulate immune responses against self-RNAs [11–13, 76–78]. As ADAR1p150 also undergoes nucleocytoplasmic shuffling, GQ-forming sequences can be targeted by this isoform in the nucleus. In this scenario, GQ binding by either Zα or Zβ localizes ADAR1 to actively transcribing genes, allowing capture of nascently transcribed RNAs and providing time for folding of ADAR dsRNA intron/exon editing substrates [49].

ZBP1 Zα1 and Zα2 also engage short GQs (U-loop-GQ_RNA_ and ALU-GQ_RNA_). In general, Zβ was consistently more responsive to variation in GQ structure than other ZBDs. These findings are notable, as they indicate that while both ADAR1 ZBDs can bind GQs, Zβ displays greater apparent structural specificity for the GQs studied. The amino-acid substitutions likely facilitate binding to the hydrophobic GQ endcaps (Supplementary Fig. S25). Zα domains also recognize GQ folds, and it is likely that binding of Zα1 and Zα2 to GQ positions ZBP1 to capture viral Z-RNAs as they fold. Otherwise, production of high-affinity GQ RNA ligands for Zα1 and Zα2 would enable viruses to inhibit ZBP1-dependent activation of cell-death pathways.

As a final layer of defense, the localization of ZBDs to stress granules is consistent with the phase separations we observe. Studies on LLPS induced by Zα domains help drive the conversion of A-RNA to Z-RNA and potentially drive anti-viral responses [79, 80]. Over time, the interactions may unwind the GQs, resulting in the formation of inter-strand hybrids that result in aggregate formation as stress granules age [68]. Although this study provides evidence for interactions between the ZBDs of both ADAR1 and ZBP1 with GQs, as well as GQ-specific binding by Zβ, the observed LLPS imposes limitations on our NMR and ITC results. However, as orthogonal methods yield similar results, we believe that our biophysical measurements are relatively agnostic to this. Several questions regarding the nature of these interactions remain. Specifically, it remains challenging to disentangle the effects of phase separation from the actual binding interactions via NMR. Several constructs result in the near-complete disappearance of resonances in NMR spectra, precluding assessment of conformational dynamics or residue specific information, and thereby limiting interpretation to a binary presence/absence of binding., Furthermore, NMR requires working at protein and nucleic acid concentrations much higher than those encountered *in vivo*, raising questions about how our findings translate to an *in vivo* setting. Nonetheless, this work provides compelling evidence of interactions between ZBDs and GQs and, more intriguingly, a potential answer to the long-standing question about the functional roles of the highly conserved ADAR Zβ domain.

## Supplementary Material

gkag419_Supplemental_File

## Data Availability

Details on quadruplex formation controls, NMR analyses of the RNA 6-mers, and ZBD binding preferences, as well as additional figures showing ITC analysis, MST analysis, NMR spectra, NMR peak heights, NMR chemical shift perturbations, and AlphaFold models. All data are available upon request.
