# Supplementary material for: Zα and Zβ domains of ADAR1 and ZBP1 bind G-quadruplexes with nanomolar affinities, establishing Zβ as a G-quadruplex-specific domain
Source: Nucleic Acids Res. 2026 May 4;54(8):gkag419. doi: 10.1093/nar/gkag419 (PMC13136903; doi:10.1093/nar/gkag419)
Supplement: gkag419_Supplemental_File [file gkag419_supplemental_file.pdf]

Supplemental Information

Zα and Zβ domains of ADAR1 and ZBP1 bind G-quadruplexes with nanomolar affinities, establishing Zβ as a G-quadruplex specific domain

Charles W. Kroft[1], Jeffrey B. Krall[1], Michael Warchol[1], Robb Welty[1], Alan Herbert[2], Morkos A. Henen[1], and Beat Vögeli[*1]

[1]Department of Biochemistry and Molecular Genetics, School of Medicine, University of Colorado Anschutz Medical Campus, Aurora, CO 80045, USA

[2]Discovery, InsideOutBio, 42 8th Street, Unit 3412, Charlestown, MA 02129, USA

The following information is supplemental and cannot be contained in the main body of the paper.

1 Analysis of nucleic acid structure

GQ nucleic acids exhibit characteristic CD spectra, wherein both parallel DNA and RNA GQ have a positive peak at 260 nm, and a negative peak at 240 nm. Antiparallel GQ (usually only formed by DNA) have positive peaks at 295 nm and 240 nm, and a negative peak at 260 nm[1]. These spectra differ from A-form RNA and ssRNA (maximum and minimum at 265-270/210-220 nm), B-form DNA and ssDNA (maximum/minimum at 275-280/245-250 nm), and Z-DNA and Z-RNA (295/260 nm)[2]. These values are described in Table S1.

By CD, we verified that each of our expected GQ sequences was folding in solution (Fig. S1A). TERRA-GQ_{RNA} showed the strongest GQ spectral characteristics, with a significant positive peak at 260 nm, and a negative one at 240 nm. As TERRA is a well-known GQ, we used this sequence to confirm that the other expected GQ RNA sequences, ALU-GQ_{RNA}

and U-loop-GQ_{RNA}, were properly folded. The CD spectrum of ALU-GQ_{RNA} closely matches that of TERRA-GQ_{RNA}, with a strong positive peak at 260 nm and a negative at 240 nm, but with a lower maximum ellipticity at 260 nm. The U-loop-GQ_{RNA} showed a similar CD pattern indicative of a folded parallel GQ, but with slightly lower maximum ellipticity than TERRA-GQ_{RNA} or ALU-GQ_{RNA}. These results indicate that we have successfully folded each of our RNA GQ sequences in GQ-promoting buffer. This is in contrast to our negative controls, TERRA-mut_{RNA} and ALU-mut_{RNA}, as their sequences cannot form GQ structures. As expected, their CD spectra show a positive peak at 275 nm, and do not have a negative peak at 240. This confirms that our GQ_{RNA} sequences are folding into parallel GQ structures while our mutated sequences are not. We also confirmed that the TTT-loop-GQ_{DNA} forms an antiparallel GQ, exhibiting characteristic positive peaks at 295 and 240 nm, and a negative peak at 260 nm (Fig. S1B).

1.1 G-quadruplex melting curves

To further characterize the folding of our RNA and DNA constructs into G-quadruplexes, we examined melting temperatures under various buffer conditions. As RNA G-quadruplexes are very stable and known to melt at temperatures upwards of 80 °C[3,4], by altering the predominant ion in solution between K^+, Na^+, and Li^+, we can visualize a difference in melting temperature (Fig. S1C). While lithium disfavors or even prevents its formation, sodium moderately and potassium strongly favor its formation[3,5].

We observed high melting temperatures for the three confirmed GQ-forming RNA constructs in KCl buffer: both ALU- and U-loop GQ_{RNA} had melting temperatures above 90 °C, and TERRA-GQ_{RNA} at 80 °C. These values are significantly higher than those of the non-GQ-forming TERRA-mut_{RNA}, which melted at 45 °C. Furthermore, the melting temperatures decreased accordingly with the GQ ion 'hierarchy' (K^+>Na^+>Li^+). ALU-GQ_{RNA} showed melting temperatures of 73 and 64 °C in NaCl/LiCl, while U-$loop_{RNA}$ demonstrated melting temperatures of 78 and 69 °C, respectively. Additionally, TERRA-GQ_{RNA} exhibited a melting temperature of 45 °C in the presence of LiCl. In contrast, TERRA-mut_{RNA} exhibited equivalent melting temperatures of 45 °C between KCl and LiCl buffers (Fig. S1C). These results confirm that each RNA GQ construct forms a G-quadruplex in solution, and that by varying the predominant ion in solution, we can alter the stability of the G-quadruplex structure.

1.2 NMR analysis of G-quadruplexes

NMR is a powerful tool orthogonal to CD spectroscopy to determine nucleic acid structure.

In the G-tetrad, there are four hydrogen bonds between the imino proton on N1, and the neighboring O6 on the neighboring guanine (Fig. S1D, circled red). These hydrogen bonds are stable and prevent hydrogen exchange with the solution, allowing characteristic G-quadruplex peaks to be visible in the 10-12 ppm region in a ^{1}H NMR spectrum. Other RNA structures in which the imino proton is visible show imino peaks further downfield (for example, hairpin RNA has imino peaks in the 13-14 ppm range)[3]. In the TERRA-mut$_{RNA}$ sequence, guanines have been swapped to cytosine, blocking the ability for G-tetrads to form and subsequently allowing N1 hydrogens to exchange with solution readily. Thus, there should be no visible peaks in the 10-12 ppm region for non-GQ RNA. Our 1D NMR analysis of the TERRA-GQ$_{RNA}$ showed strong peaks in the 10-12 ppm range, further confirming its ability to form a GQ structure, while the TERRA-mut$_{RNA}$ had no visible peaks in this range (Fig. S1D).

We also analyzed ALU- and U-loop-GQ$_{RNA}$ via ^{1}H NMR. Both of these constructs exhibited peaks in the GQ imino region, but with slightly broader peaks than seen with TERRA-GQ$_{RNA}$. This could be a result of a less stable GQ structure, but still indicates the presence of a G-quadruplex. Subsequently, we also examined the effects of altering the predominant buffer ion via NMR. In accordance with our melting results, we observe that for both ALU- and U-loop-GQ, the buffer condition reduces the stability/degree of folded GQ. We see that in high Na^{+} buffer, the imino peaks shift slightly downfield and begin to experience some peak broadening. Furthermore, in Li^{+} buffer, the peaks remain at the same position as Na^{+}, but are even broader (Fig. S1). This suggests that not only do we decrease the stability of GQ, but we also shift the equilibrium in solution towards a more unfolded state. We also examined the temperature stability of ALU-GQ$_{RNA}$ in various buffers. In K^{+} buffer, there are no visible changes to the imino peaks between 35-75°C. In contrast, in Na^{+} buffer, we begin to see peak intensity reductions as temperature increases, and the complete disappearance of imino peaks in Li^{+} (Fig. S2).

Taking the CD, temperature melting and NMR data together, we confirmed that all our constructs of interest form G-quadruplexes under our experimental *in vitro* conditions. Placing G-quadruplexes in either KCl, NaCl, or LiCl buffer exhibits variable stability depending on the predominant ion in the buffer, with K^{+} having the strongest GQ-promoting effects, and Li^{+} the weakest. Thus, we can utilize these modulations to characterize GQ stability and to study if Zα and Zβ domains exhibit differential binding to the same RNA sequence depending on its secondary structure.

Table S1: Comparison of characteristic maximum and minimum CD ellipticity values for different DNA and RNA secondary structures.

	**Positive peak (nm)**	**Negative peak (nm)**	**Construct**
A-RNA, ssRNA	265-270	210-220	TERRA-mut$_{RNA}$, ALU-mut$_{RNA}$
B-DNA, ssDNA	275-280	245-250	-
Z-DNA, Z-RNA	295	260	-
Antiparallel G-quadruplex	295,240	260	TTT-loop-GQ$_{DNA}$
Parallel G-quadruplex	260	240	TERRA-GQ$_{RNA}$, ALU-GQ$_{RNA}$, U-loop-GQ$_{RNA}$

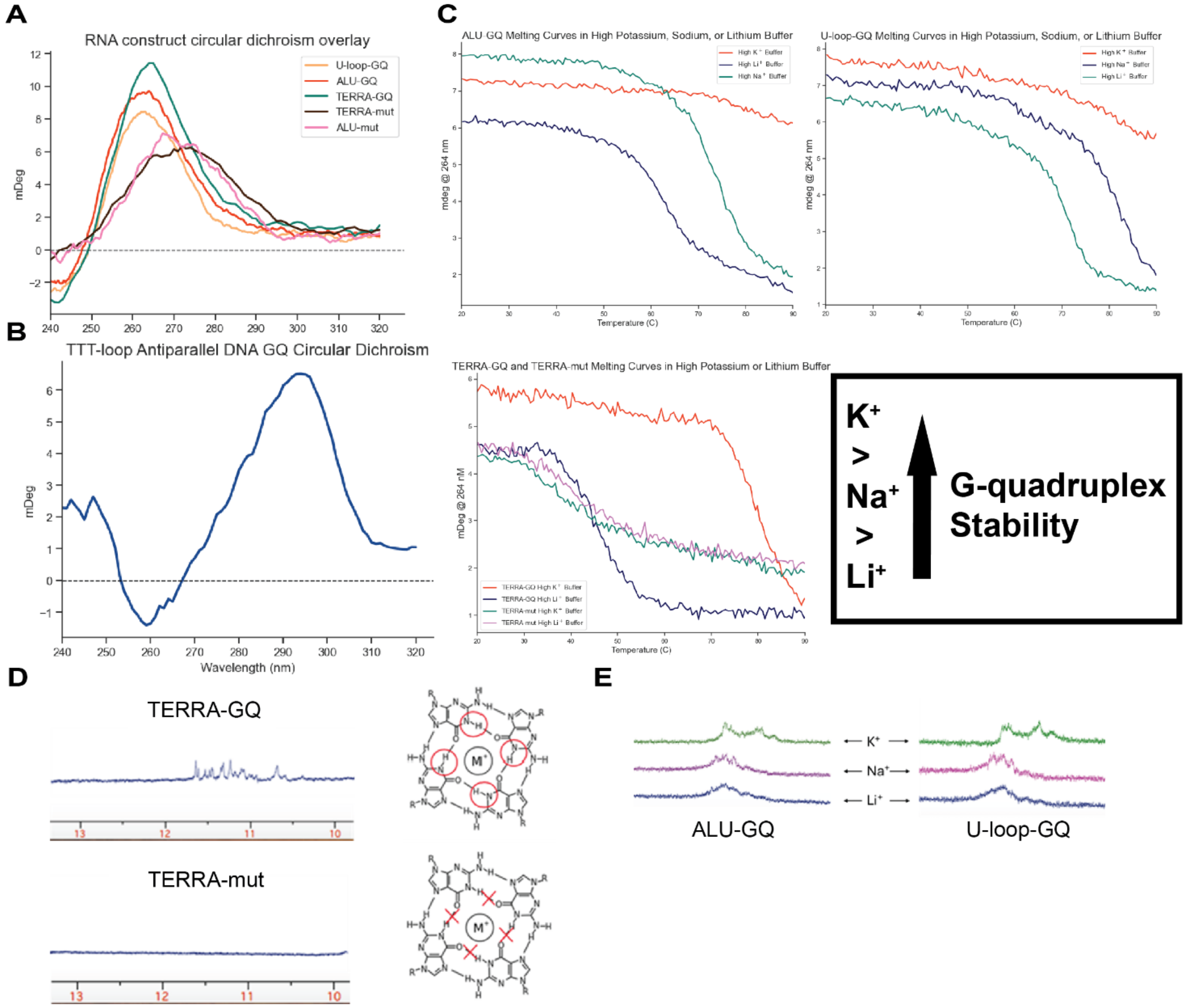

Figure S1: **Assessment of G-quadruplex formation of RNA and DNA constructs**. A) Overlaid CD spectra of RNA constructs, all constructs except TERRA-mut$_{RNA}$ and ALU-mut$_{RNA}$ adopt a parallel GQ structure. B) CD spectrum of TTT-loop-GQ$_{DNA}$ shows peaks typical of antiparallel GQ. C) CD melting curves of ALU-GQ$_{RNA}$ and U-loop-GQ$_{RNA}$ in KCl, NaCl, or LiCl buffer, and TERRA-GQ$_{RNA}$ and TERRA-mut$_{RNA}$ in KCl or LiCl buffer at 35 °C. D) Imino region ^{1}H NMR shows GQ peaks for TERRA-GQ$_{RNA}$ but no peaks for TERRA-mut$_{RNA}$. E) Comparison of ^{1}H NMR imino peaks for ALU-GQ$_{RNA}$ and U-loop-GQ$_{RNA}$ in KCl, NaCl, or LiCl buffer at 35 °C.

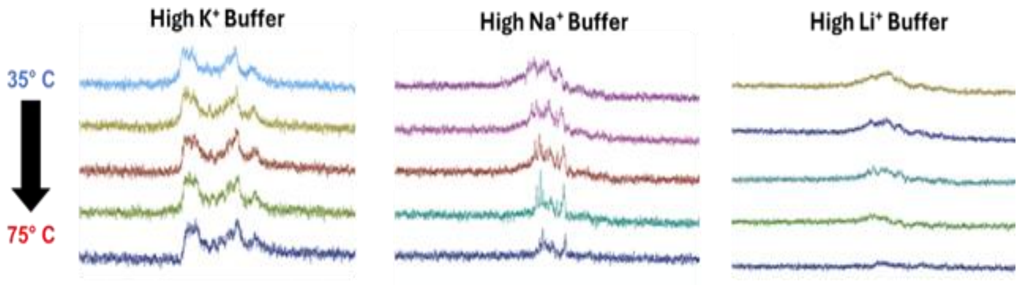

Figure S2: **G-quadruplex melting by NMR.** U-loop-GQ_{RNA} in KCL, NaCl, or LiCl buffer at 35, 45, 55, 65, and 75 °C. Temperature has no visible effect in the K^+ buffer, while Na^+ exhibits mild temperature sensitivity, as evidenced by peak intensity reduction; the GQ signal is completely abrogated at 75°C in the Li^+ buffer.

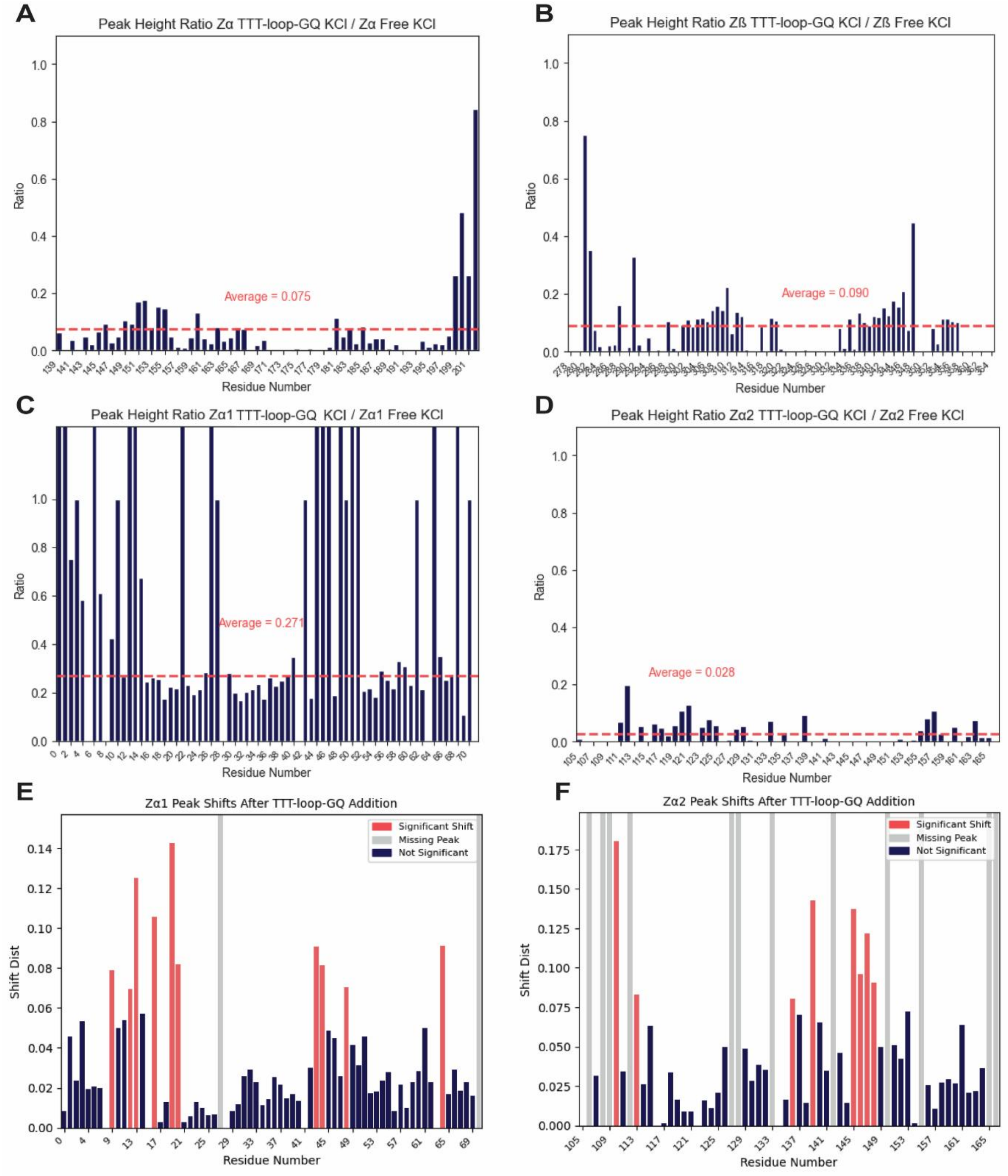

Figure S3: **HSQC peak height ratio plots of ADAR1 and ZBP1 ZBDs binding to TTT-loop-GQ_{DNA}, CSP plots of binding to Zα1 and Zα2.** Peak ratio plots are represented as a ratio of bound (nucleic acid + protein) to free protein. The red line indicates the mean. A-D) ADAR1 Zα, Zβ, ZBP1 Zα1, Zα2 + TTT-loop-GQ_{DNA}, respectively. E, F) CSP plots binding with Zα1 and Zα2, respectively.

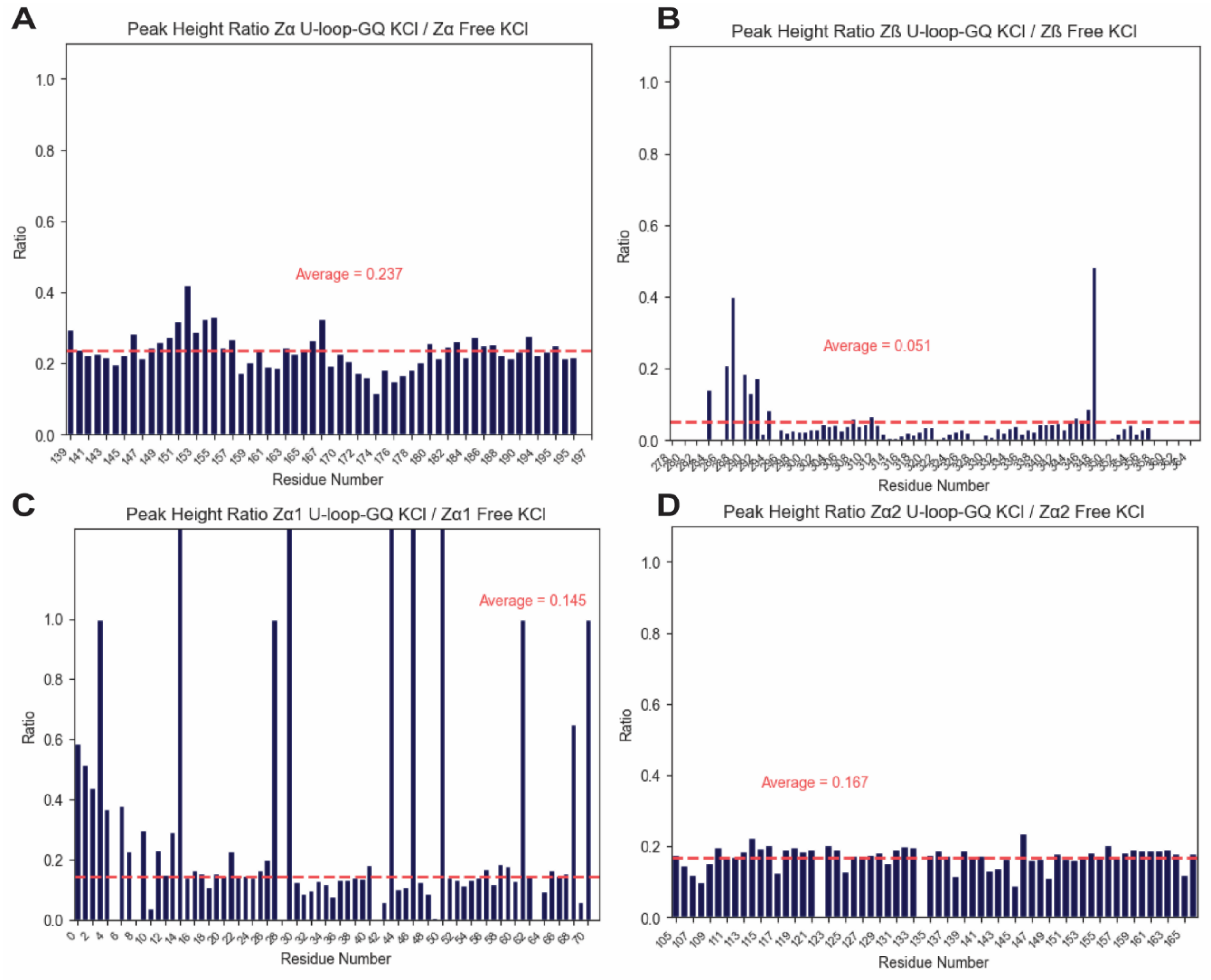

Figure S4: **HSQC peak height ratio plots of ADAR1 and ZBP1 ZBDs binding to U-loop-GQ$_{RNA}$.** Plots are represented as a ratio of bound (nucleic acid + protein) to free protein. The red line indicates the mean. A-D) ADAR1 Zα, Zβ, ZBP1 Zα1, Zα2 + U-loop-GQ$_{RNA}$, respectively.

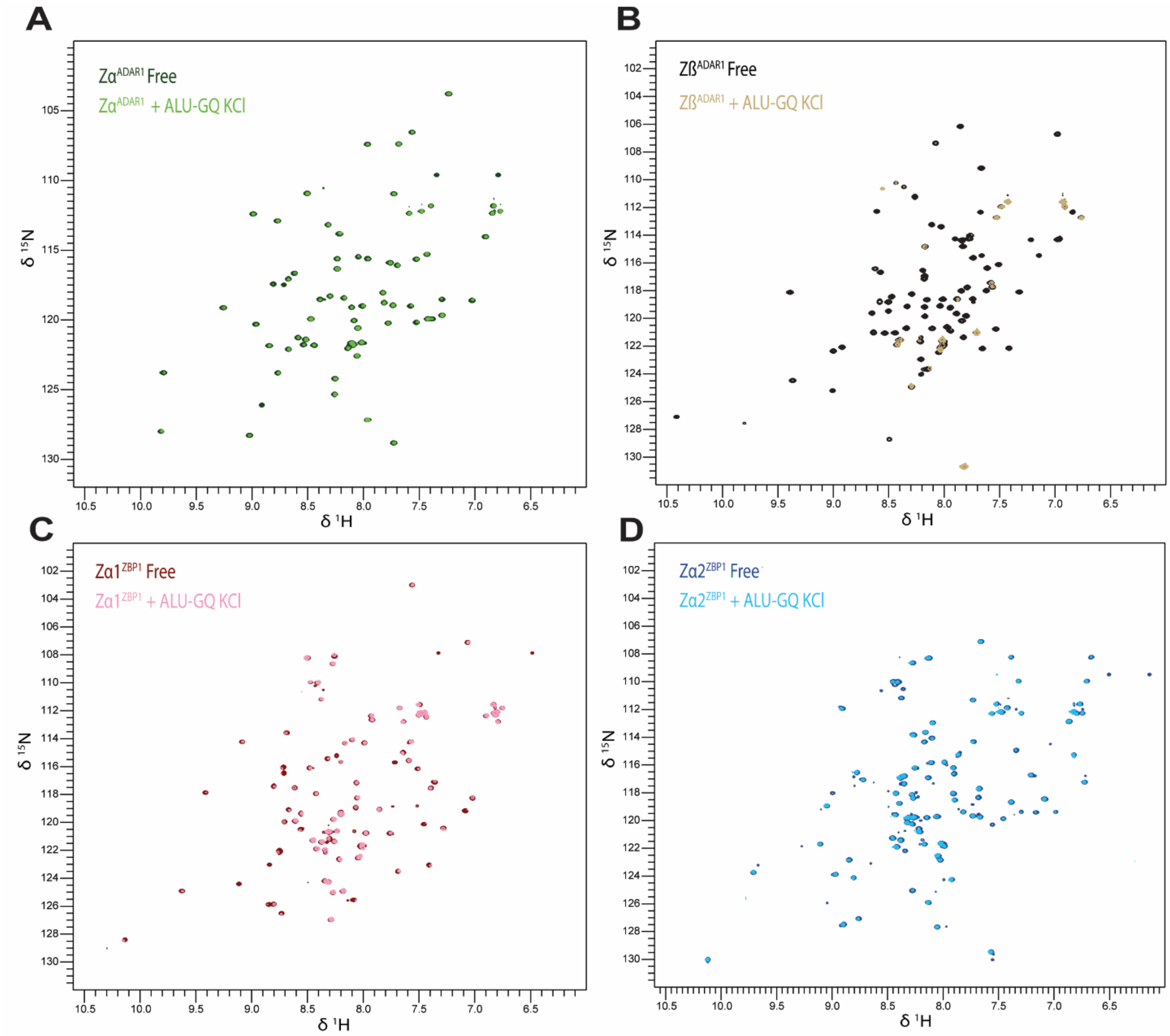

Figure S5. **Zα and Zβ domains interact with ALU-GQ$_{RNA}$**. Overlaid 2D HSQC spectra of ALU-GQ$_{RNA}$in the presence of A) Zα domain of ADAR1, B) Zβ domain of ADAR1, C) Zα1 domain of ZBP1, and D) Zα2 domain of ZBP1.

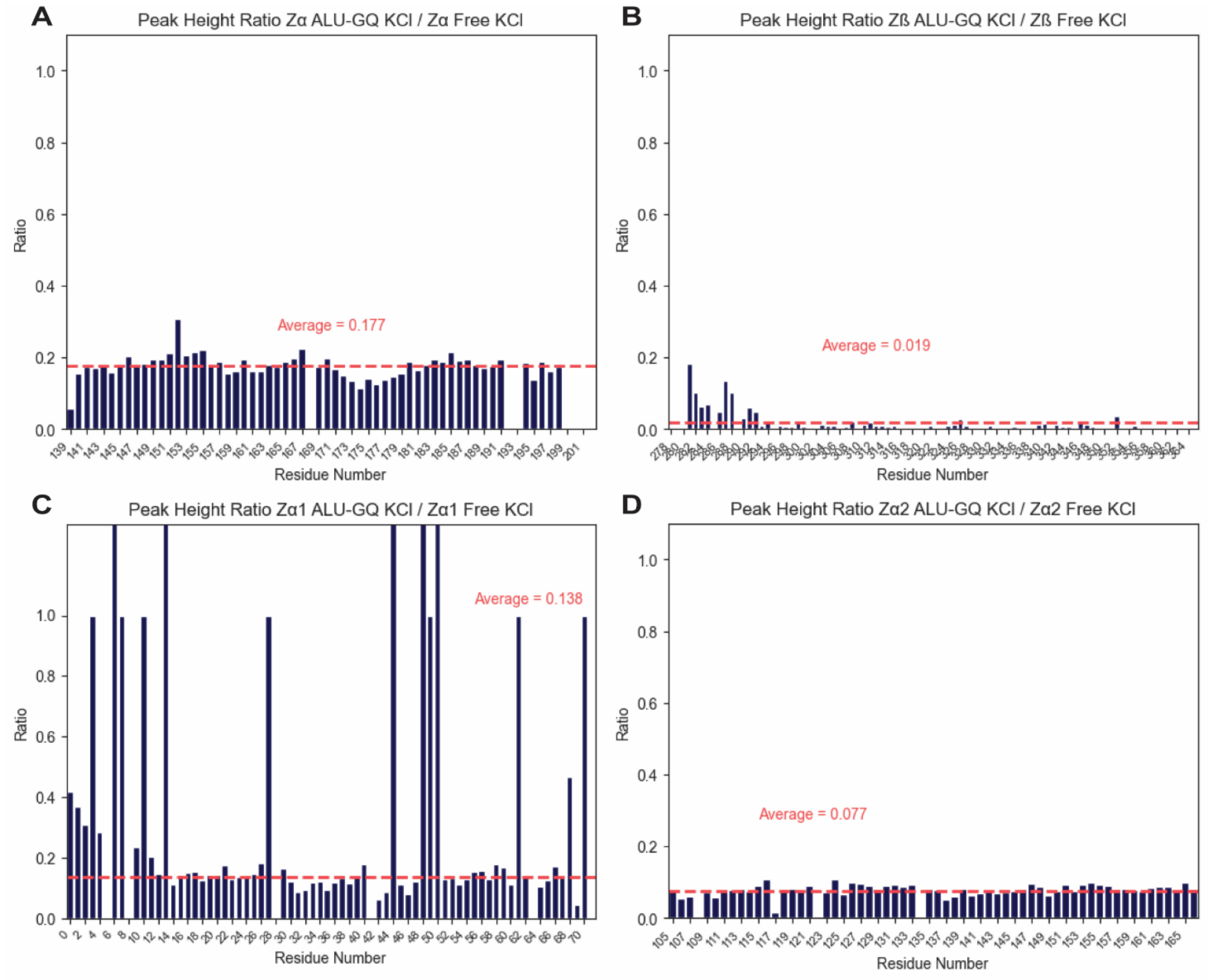

Figure S6: **HSQC peak height ratio plots of ADAR1 and ZBP1 ZBDs binding to ALU-GQ$_{RNA}$.** Plots represented as a ratio of bound (nucleic acid + protein)/free protein. The red line indicates the mean. A-D) ADAR1 Zα, Zβ, ZBP1 Zα1, Zα2 + ALU-GQ$_{RNA}$, respectively.

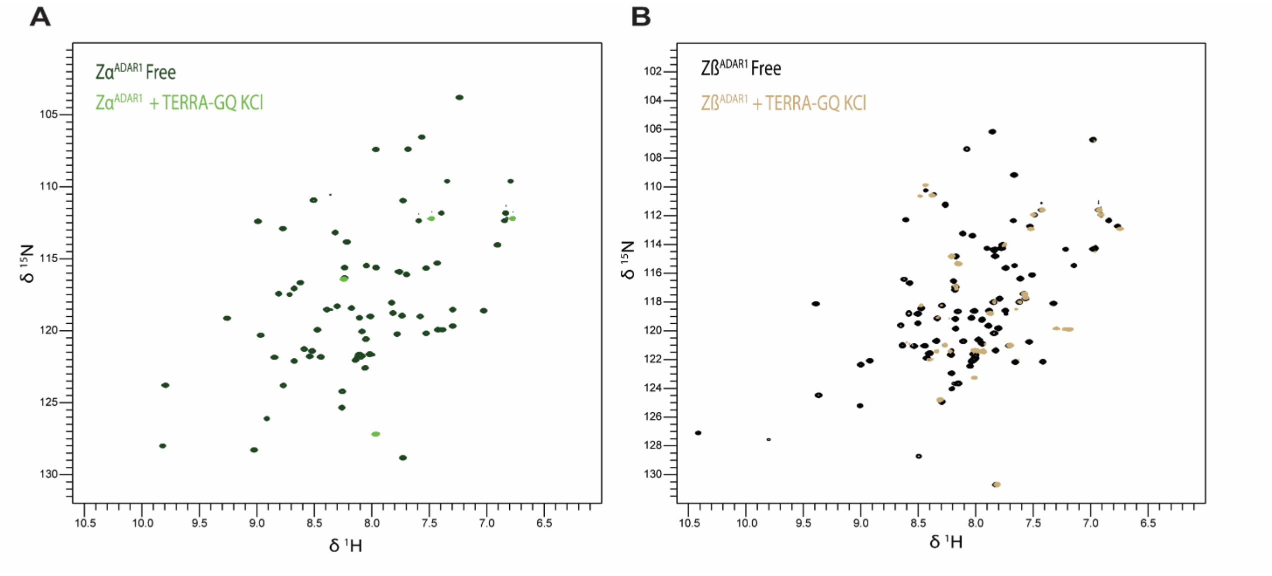

Figure S7: **Comparison of interaction of ADAR1 Zα and Zβ subunits with TERRA-GQ$_{RNA}$**. Overlaid 2D HSQC spectra of free or bound protein of A) free Zα ADAR1 free and in the presence of TERRA-GQ$_{RNA}$, B) free Zβ ADAR1 free and in the presence of TERRA-GQ$_{RNA}$.

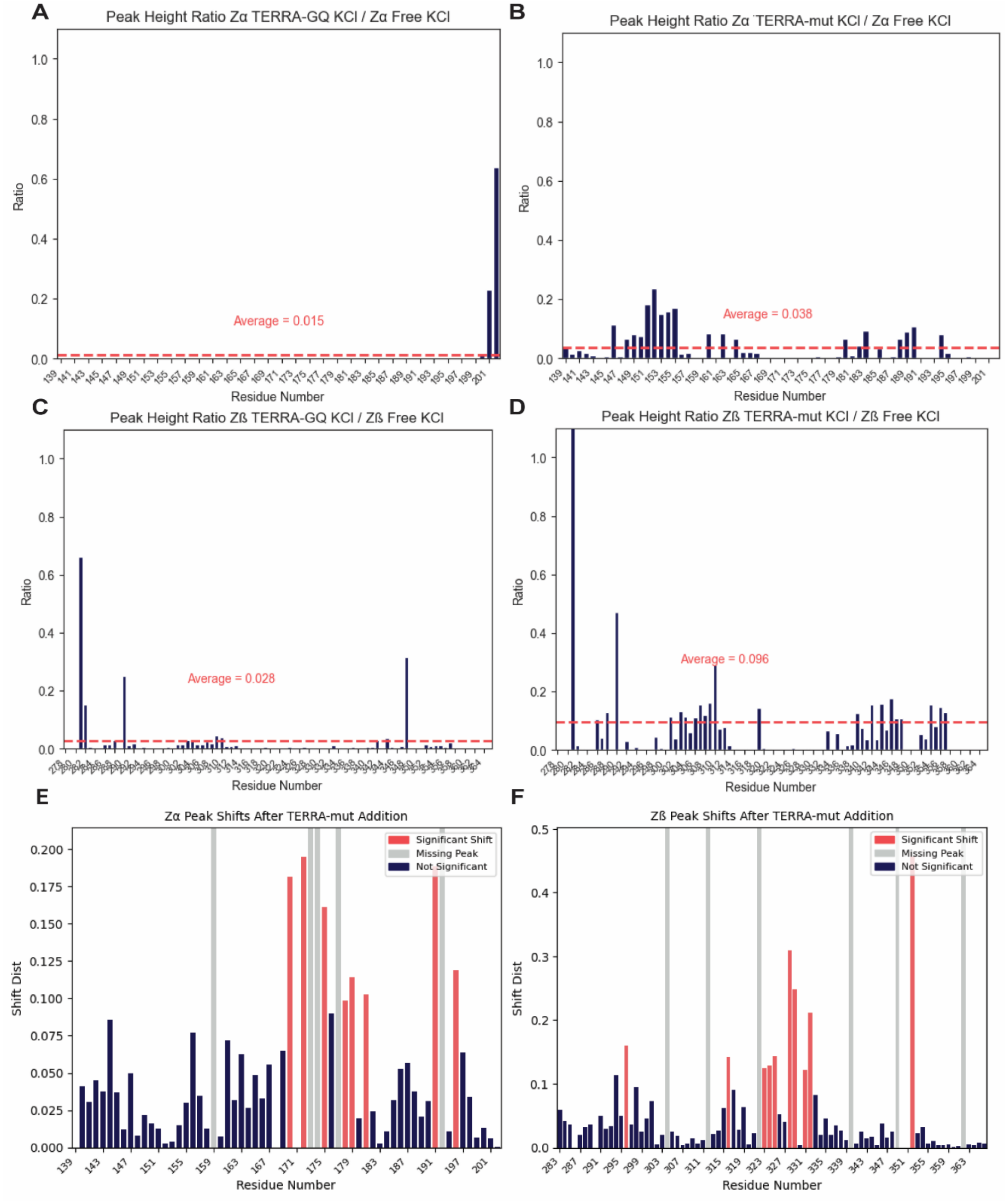

Figure S8: **HSQC peak height ratio plots and CSP plots of ADAR1 Zα and Zβ binding to TERRA-GQ$_{RNA}$ and TERRA-mut$_{RNA}$.** Plots are represented as a ratio of bound (nucleic acid + protein)/free protein. A, B) Peak height ratios of Zα + TERRA-GQ$_{RNA}$ or TERRA-mut$_{RNA}$. C, D) Peak height ratios of Zβ + TERRA-GQ$_{RNA}$ or TERRA-mut$_{RNA}$. E, F) CSPs of Zα and Zβ binding to TERRA-mut$_{RNA}$, respectively.

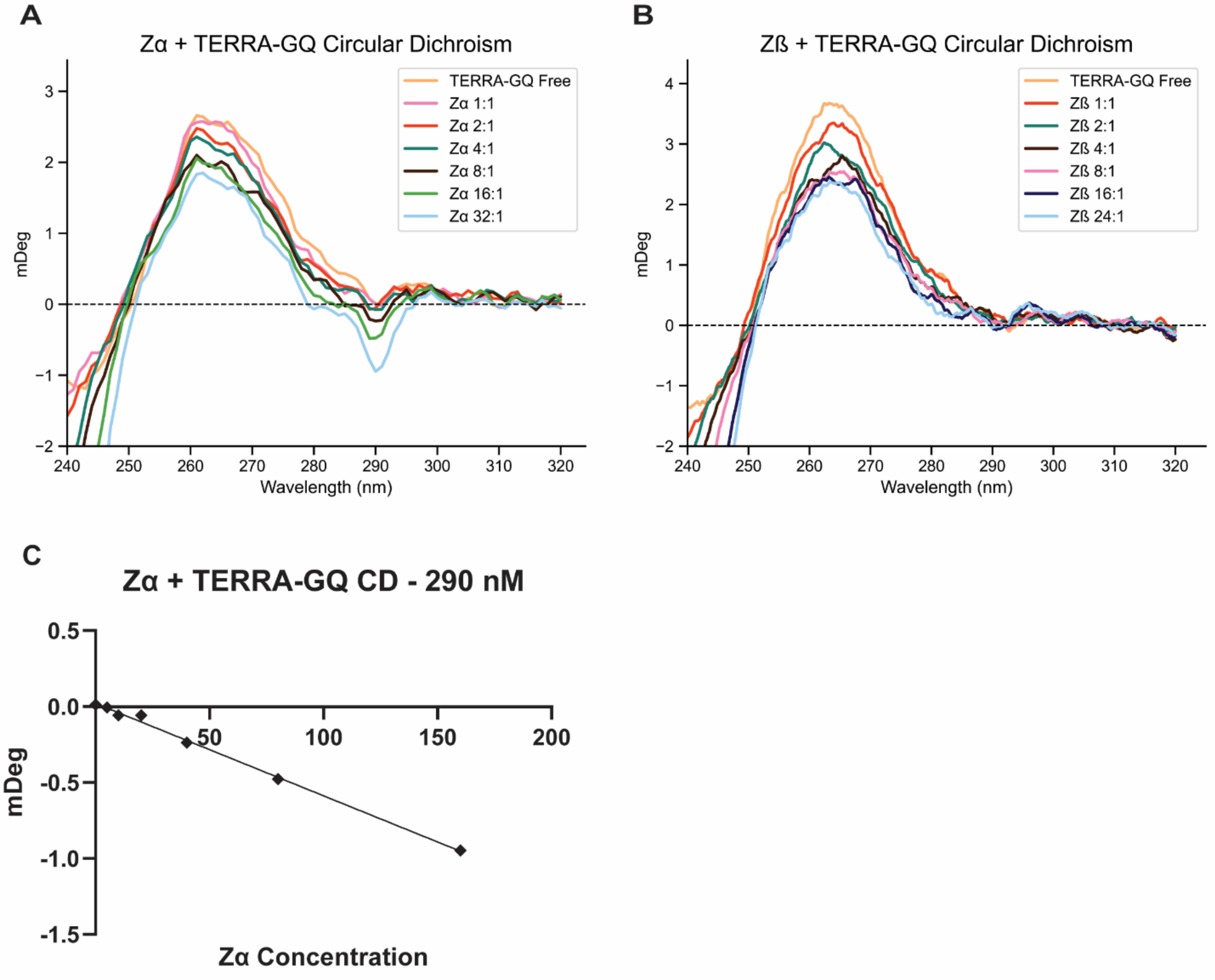

Figure S9**. Circular dichroism spectra of TERRA-GQ** with A) Zα added at a ratio of 1:1 to 32:1 protein:nucleic acid, B) Zβ added at a ratio of 1:1 to 24:1 protein:nucleic acid, and C) CD signal vs Zα concentration at 290 nM, showing linear change with protein concentration.

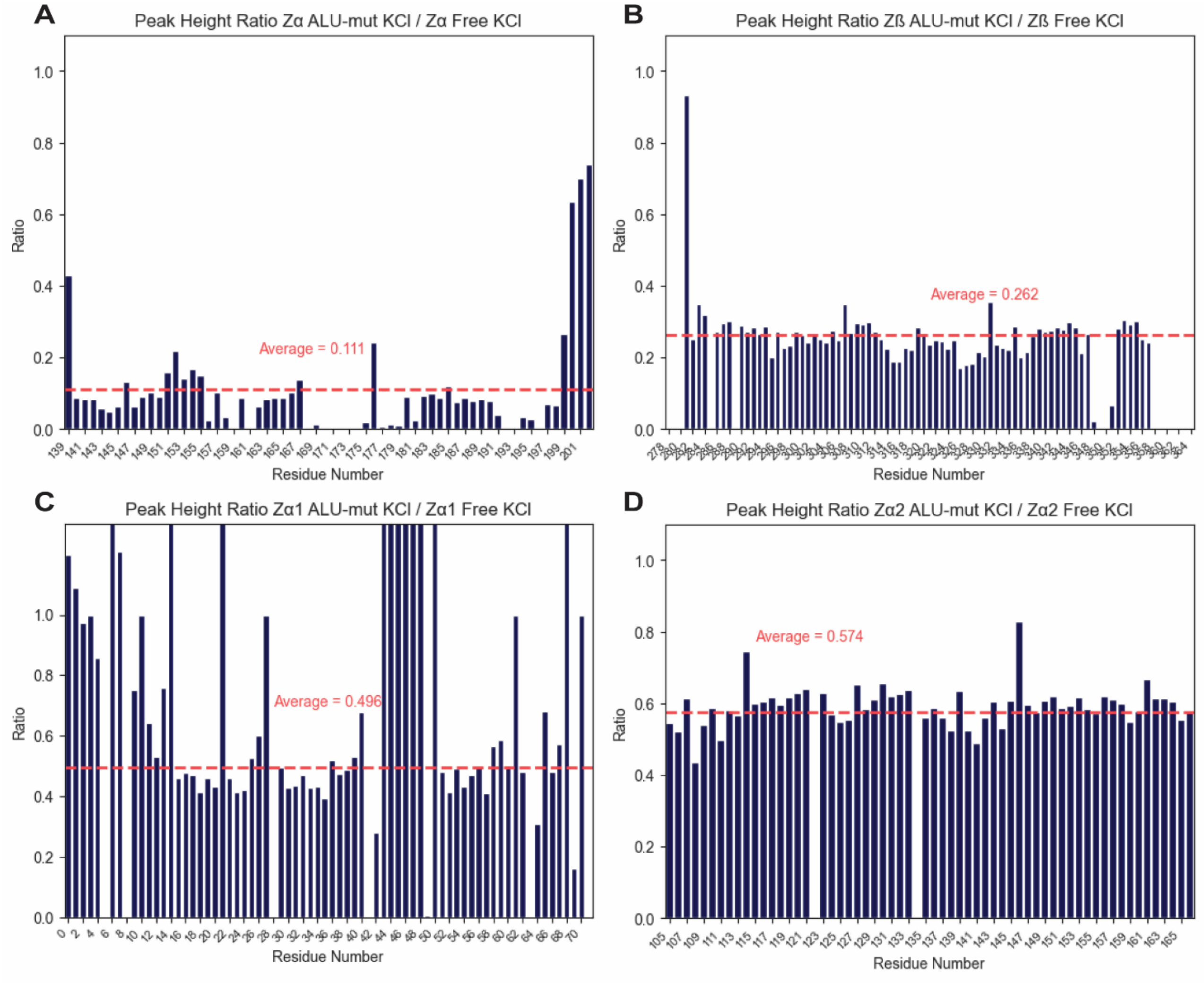

Figure S10: **HSQC peak height ratio plots of ADAR1 and ZBP1 ZBDs binding to ALU-mut$_{RNA}$.** Plots are represented as a ratio of bound (nucleic acid + protein)/free protein. The red line indicates the mean. A-D) ADAR1 Zα, Zβ, ZBP1 Zα1, Zα2 + ALU-mut$_{RNA}$, respectively.

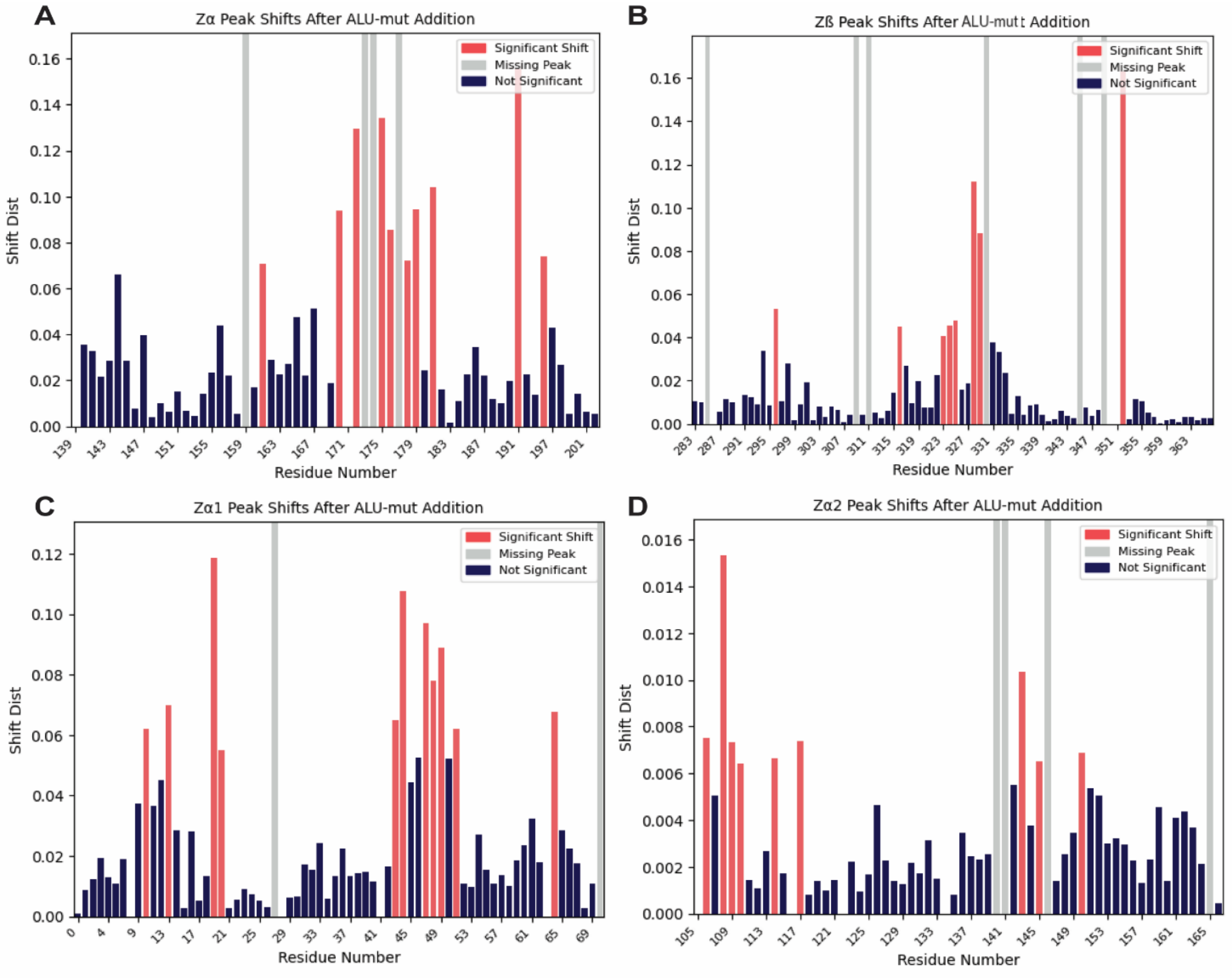

Figure S11: **Chemical shift perturbation plots of ADAR1 and ZBP1 ZBDs binding to Alu-mut$_{\mathbf{RNA}}$.** A-D) CSPs of ADAR1 Zα, Zβ, ZBP1 Zα1, and Zα2 binding to Alu-mut$_{\text{RNA}}$, respectively.

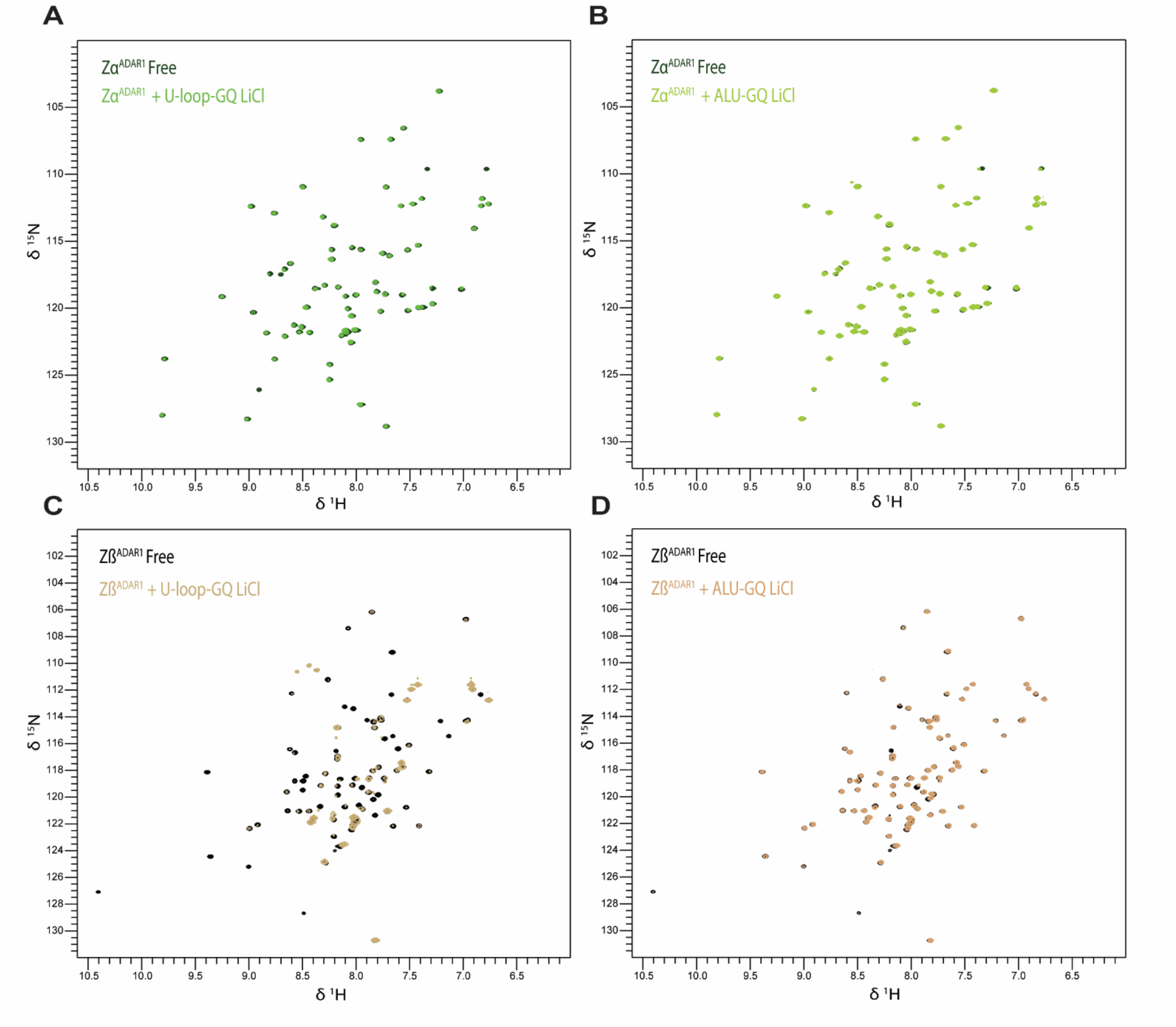

Figure S12: **Comparison of ADAR1 ZBD binding to U-loop-GQ$_{RNA}$ and ALU-GQ$_{RNA}$ in LiCl buffer.** Overlay of HSQC spectra in high Li^{+} of Zα domain in complex with A) ALU-GQ$_{RNA}$ and B) U-loop-GQ$_{RNA}$. Overlay of HSQC spectra in high Li^{+} of Zβ domain in complex with C) ALU-GQ$_{RNA}$ and D) U-loop-GQ$_{RNA}$.

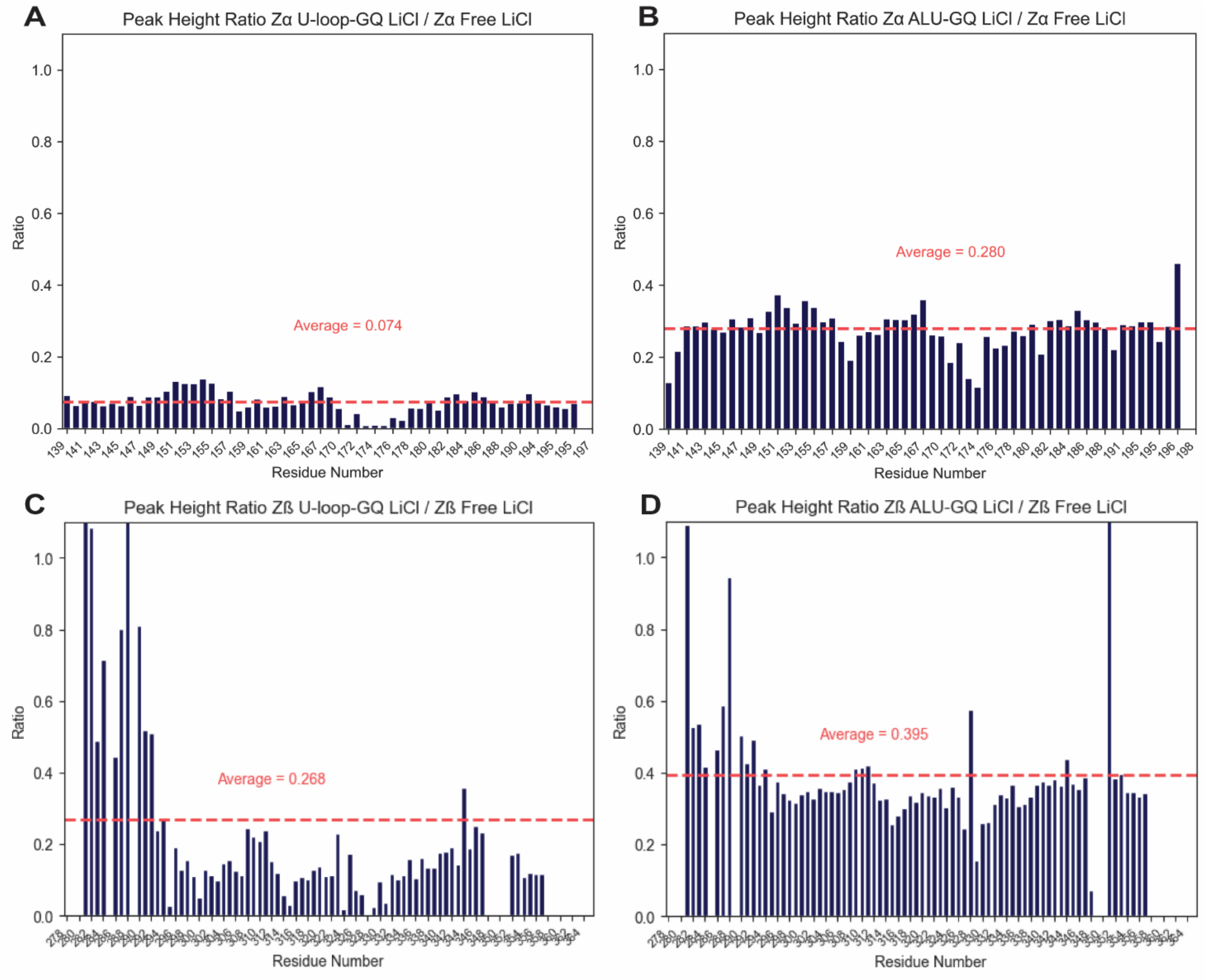

Figure S13: **HSQC peak height ratio plots of ADAR1 Zα and Zβ binding to U-loop-GQ and ALU-GQ$_{RNA}$ in Li^{+} buffer.** Plots are represented as a ratio of bound (nucleic acid + protein)/free protein. A, B) Zα + U-loop-GQ$_{RNA}$ and ALU-GQ$_{RNA}$, respectively. C, D) Zβ + U-loop-GQ$_{RNA}$ and ALU-GQ$_{RNA}$, respectively.

2 ADAR1 Zβ binds preferentially to an intermolecular G-quadruplex compared to its individual strands

To investigate if the observed binding to G-quadruplexes was a result of guanine

base preference, we tested binding with 6mer derivates of either TERRA-GQ_{RNA} (UUAGGG) or TERRA-mut_{RNA} (UUACCG). Both UUAGGG and UUACCG are single stranded at low concentrations, allowing direct measurement of G versus C specificity. Further, we hypothesized that at higher concentrations UUAGGG could form an intermolecular GQ (ssUUAGGG; 30 uM vs GQ-UUAGGG; 100 uM) (Fig. S14A, B). The ability to control formation of GQ in solution by adjusting concentration would allow us to directly examine the structural specificity of the ZBDs by eliminating any variables regarding sequence or buffer condition.

We confirmed the formation of an intermolecular GQ at 100 μM via 1D ^{1}H NMR, where UUAGGG showed similar imino peaks as TERRA-GQ_{RNA}. On the other hand, UUACCG and UUAGGG at 30 μM displayed no imino peaks, indicative of predominantly single stranded nature (Fig. S15B). CD indicates partial GQ population at 30 μM, but NMR suggests that this is a low population state, potentially lower than 10%. Additionally, neither Zα nor Zβ underwent phase separation upon addition at either low or high concentration; this could indicate LLPS being a NA length dependent effect or even a GQ structural effect.

We collected HSQC spectra of Zα with UUACCG at 30 and 100 μM (Fig. S15A). At the lower concentration, Zα showed minor peak shifts (Fig. S14C). The magnitude of these shifts was greater at higher concentration. Zα also showed peak shifts with UUAGGG at 30 μM (Fig. S15B), with a magnitude of peak shifts comparable to that of UUACCG at 100 μM. This indicated stronger binding to ssUUAGGG than ssUUACCG. At 100 μM UUAGGG (GQ-UUAGGG), Zα exhibited nearly equivalent CSPs as with ssUUAGGG (Figs. S14D, S15B). The magnitude of CSPs at 30 μM are greater than what would be expected for binding to the low fraction of GQ in this sample, suggesting that Zα also interacts with ssUUAGGG. The overall CSP trend indicates that the binding mode is similar between these two structural states. Peak height ratio plots showed a greater reduction with GQ-UUAGGG than the other three measurements (Fig. S16A-D). This could be attributed to a Zα preference for GQ, or may also be simply a result of increased UUAGGG concentration. Overall, these results indicate that Zα may in fact have a slight guanine base preference but it is difficult to fully disentangle differences between ss and GQ binding.

With UUACCG, Zβ displayed slight peak shifts that were comparable at both the 30 and 100 μM concentration points (Figs. S14E, S15C). Peak height reductions were also minimal (Figs. S16E, F). With ssUUAGGG, Zβ displayed very minimal peak shifts and is likely binding very weakly (Figs. S14F, S15D). The magnitude of these shifts was significantly lower than at either UUACCG concentration point, suggesting that Zβ may even have preference for cytosine over guanine. With GQ-

UUAGGG, Zβ displayed much larger CSPs than with UUACCG or ssUUAGGG (Figs. S14F, S15D). Peak height reduction plots indicate a similar preference (Figs. S16G, H). These results strongly indicate that Zβ has no sequence preference for guanine but instead has strong GQ structural preference.

We have previously published electrophoretic mobility shift assays (EMSA) demonstrating binding of ADAR1 Zα to a γ32P-labeled $r(CpG)_8$ construct (Nichols et al., 2024, JACS, 146, 677, Figure S28 in the Supporting Information)[6]. 50% of this RNA consists of guanine. The RNA concentration was 24 pM, a condition where duplexes are not stably formed and single stranded species can be probed. Addition of Zα domain first increases the single-stranded population, indicating its role in promoting melting, before causing complex formation (thereby flipping the RNA into double-stranded Z-form) at higher concentration. We also observed this initial promotion of single-stranded RNA formation when we added Zβ domain or a Zα mutant that is unable to flip RNA to Z-form. This is in agreement with the current observations that ZBDs can bind ssRNA.

Altogether, our measurements indicate that both Zα and Zβ are potent GQ binders, but Zβ may have a stricter GQ structural preference than Zα.

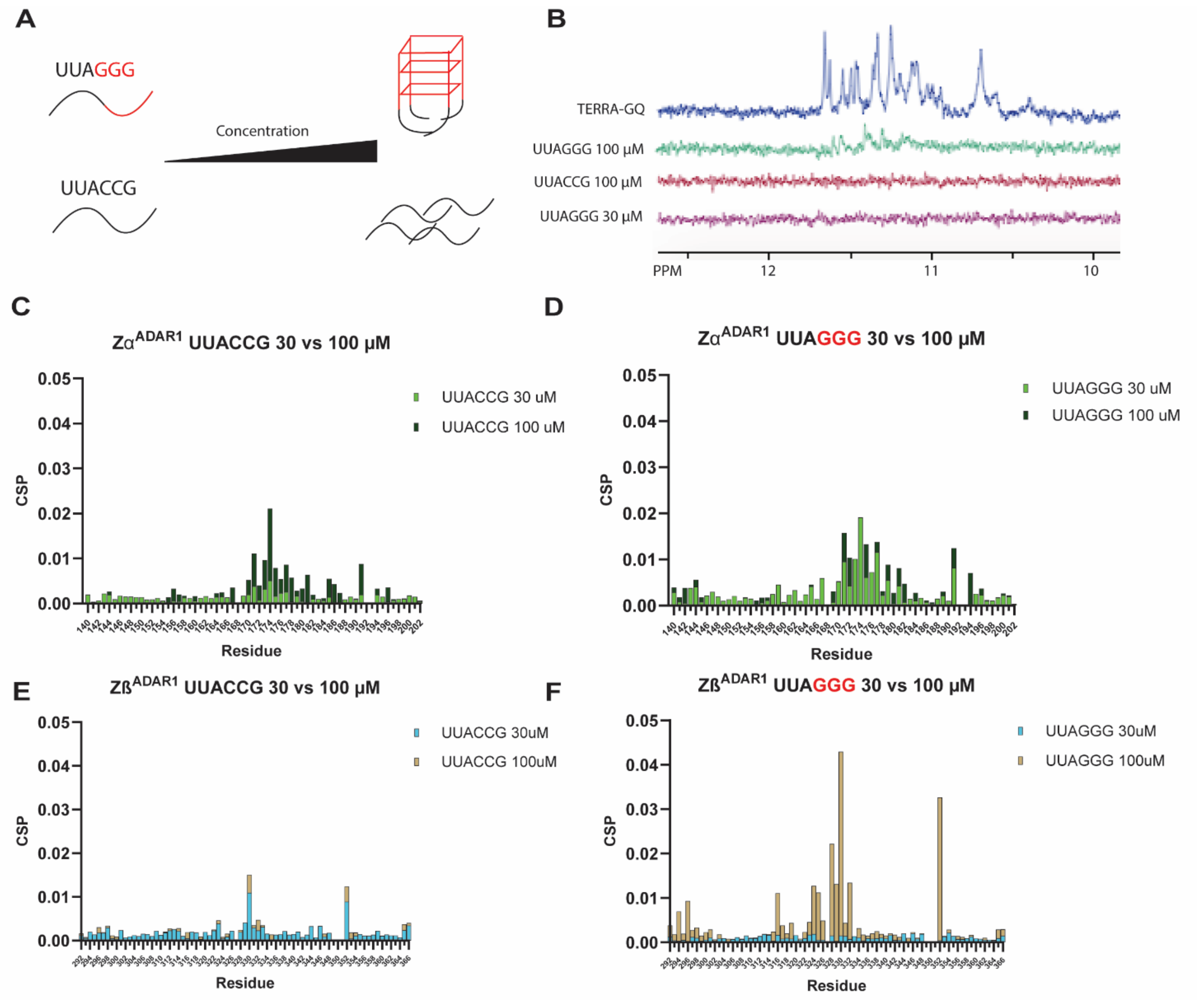

Figure S14**: ADAR1 Zα and Zβ domain binding comparison between UUAGGG and UUACCG 6mers at low and high concentration**. A) Model describing the intermolecular interactions at low and high concentration for both 6mers. B) 1D ^{1}H NMR spectra comparing UUACCG to UUAGGG at low and high concentrations, as well as comparison to TERRA-GQ. Overlaid CSP plots of Zα bound to C) UUACCG and D) UUAGGG at 30 and 100 µM. Overlaid CSP plots of Zβ bound to E) UUACCG and F) UUAGGG at 30 and 100 µM.

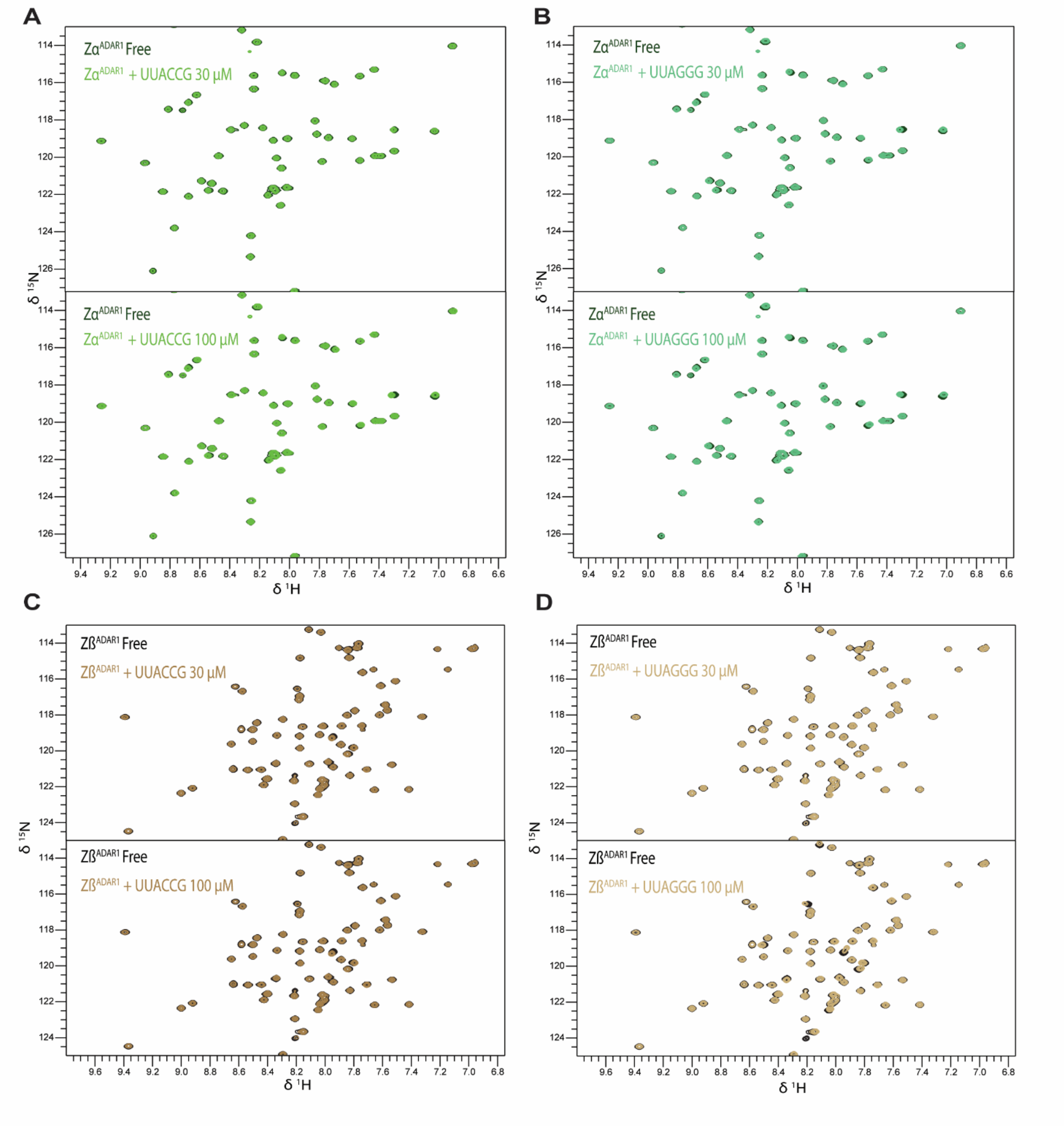

Figure S15. **ADAR1 Zα and Zβ domains HSQC with UUACCG or UUAGGG 6mer**. Overlaid 2D HSQC free protein or interacting with A) Zα of ADAR1 with 30 µM UUACCG (top) or 100 µM UUACCG (bottom), B) Zα of ADAR1 with 30 µM UUAGGG (top) or 100 µM UUAGGG (bottom), C) Zß of ADAR1 with 30 µM UUACCG (top) or 100 µM UUACCG (bottom), and D) Zß of ADAR1 with 30 µM UUAGGG (top) or 100 µM UUAGGG (bottom).

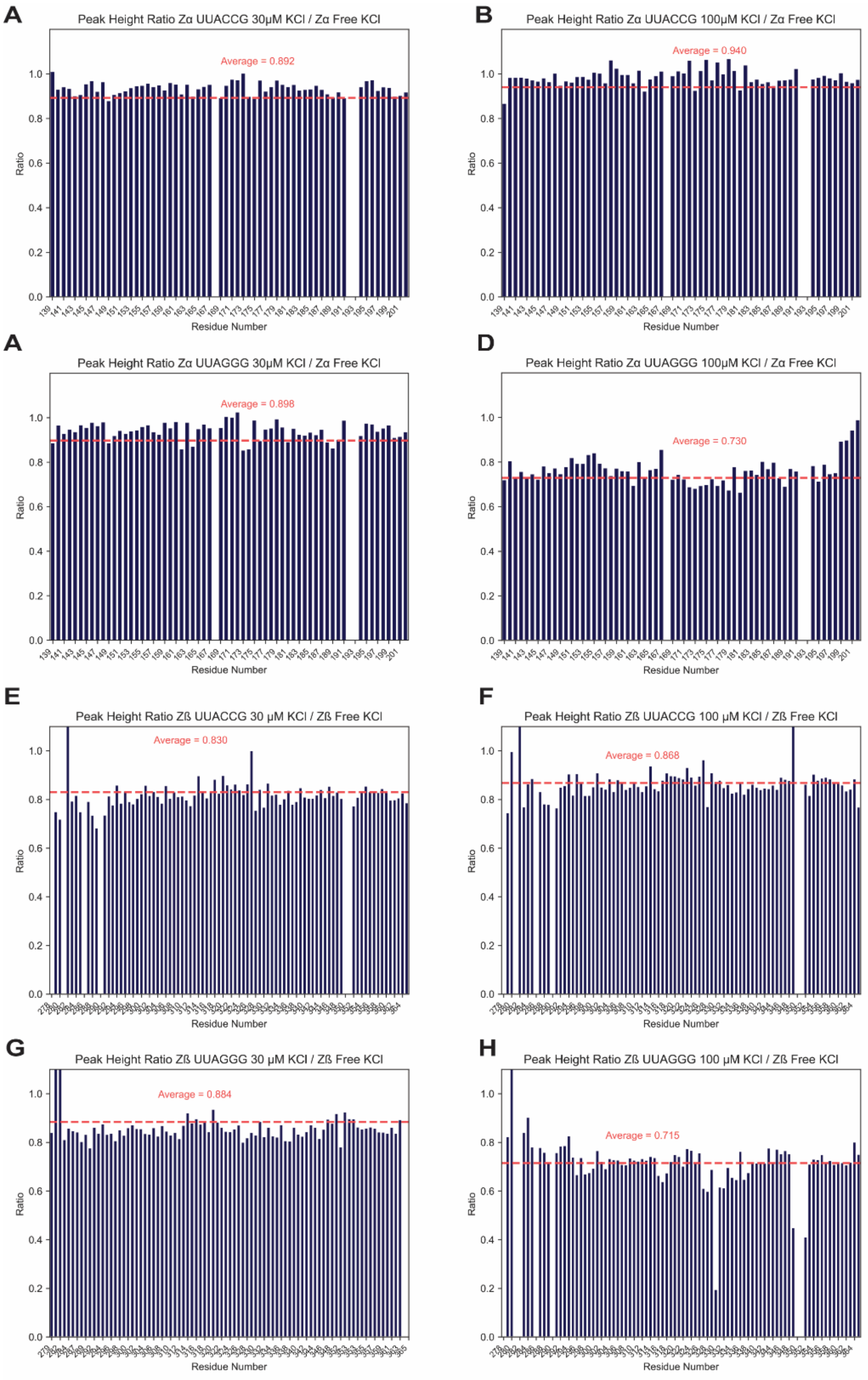

Figure S16. **Peak height ratio plots of Zα and Zβ of ADAR1 with UUACCG and UUAGGG 6mers.** Zα with A) 30 µM UUACCG and B) 100 µM UUACCG. Zα with C) 30 µM UUAGGG and D) 100 µM UUAGGG. Zß with E) 30 µM UUACCG and F) 100 µM UUACCG. Zβ with G) 30 µM UUAGGG and H) 100 µM UUAGGG.

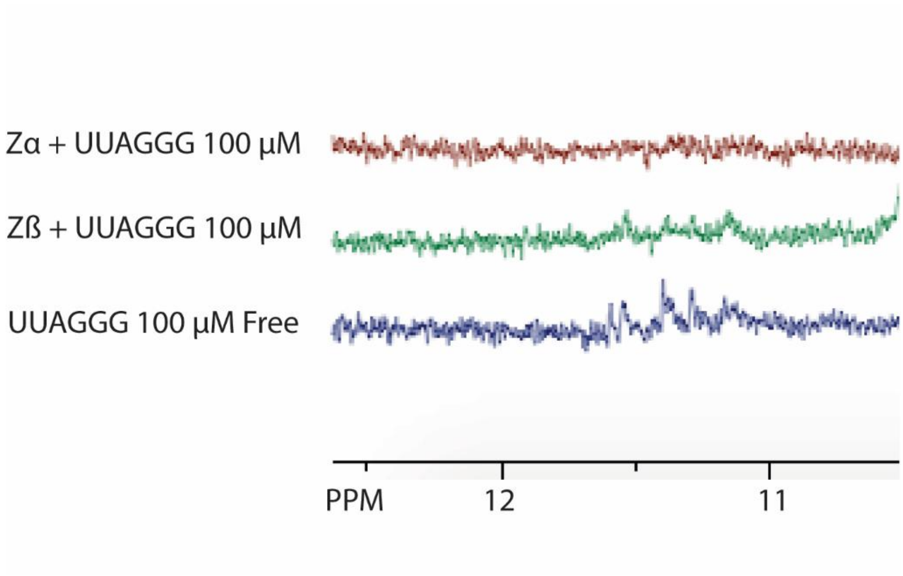

Figure S17. **Imino region of 1D ^{1}H NMR spectra overlay of Zα and Zβ domains bound to intermolecular UUAGGG GQ compared to free UUAGGG.**

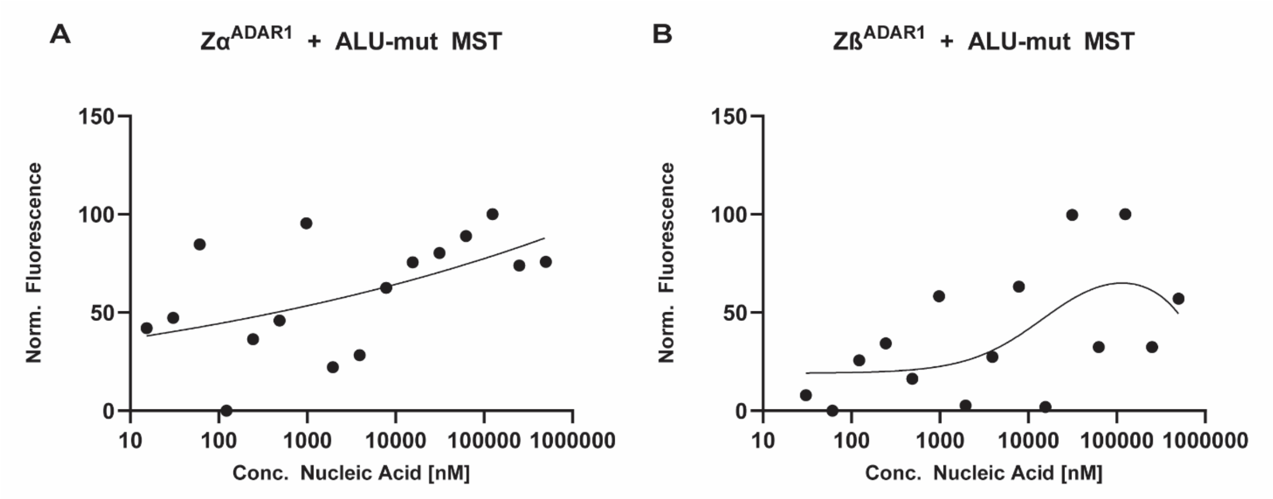

Figure S18**. 1.5 s MST data of ALU-mut$_{\mathbf{RNA}}$** interacting with A) Zα and B) Zβ showing lack of a binding curve.

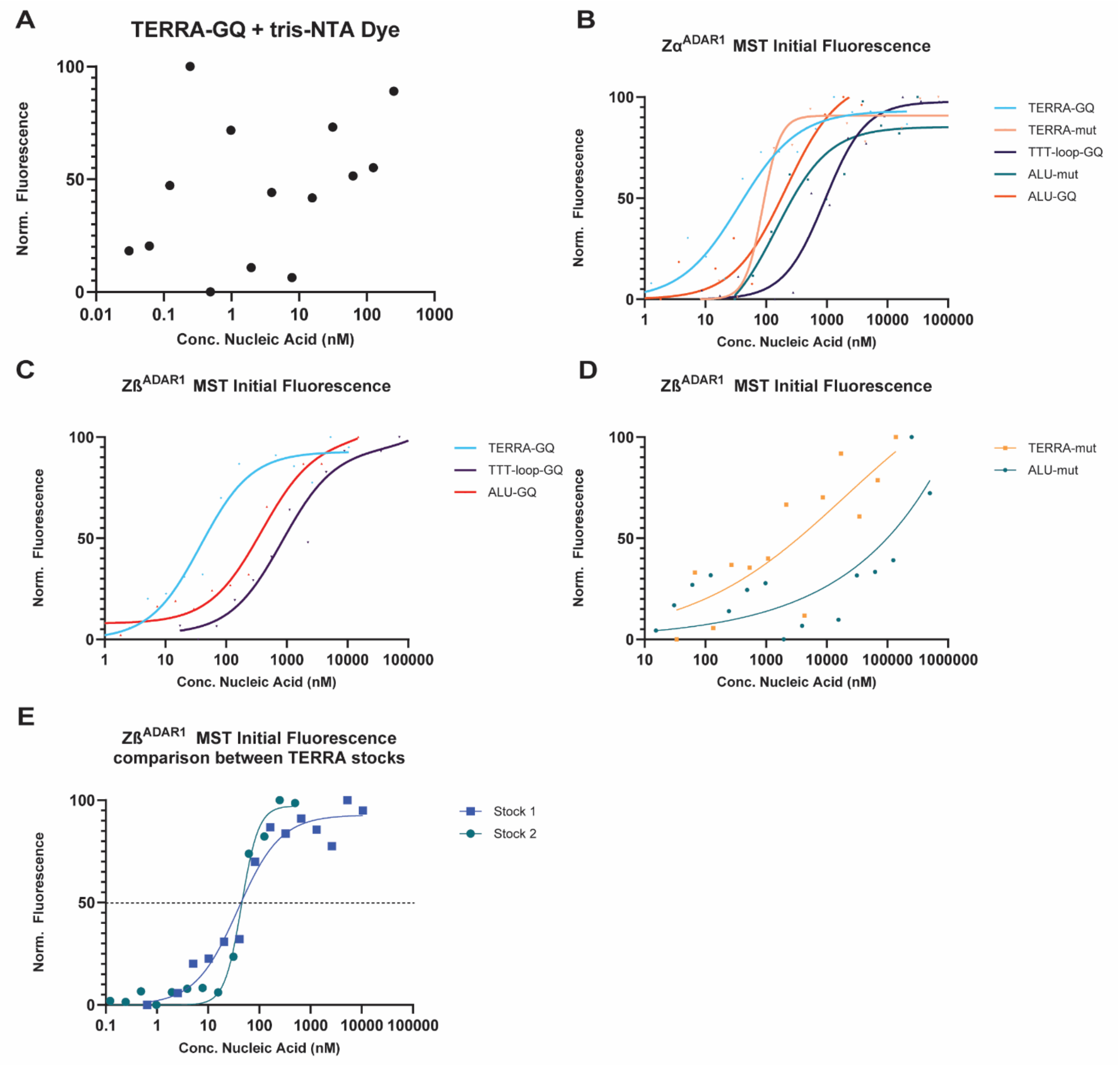

Figure S19: **Initial fluorescence measurements of ADAR1 Zα and Zβ.** A) TERRA-GQ titrations in presence of NTA dye but not protein. B) Zα initial fluorescence curve fits with various nucleic acids. C) Zβ initial fluorescence curve fits with TERRA, TTT-loop, and ALU G-quadruplexes. D) Zβ initial fluorescence curve fits of TERRA and ALU mutant sequences. E) Zβ comparison between two independently generated TERRA-GQ$_{RNA}$ stocks.

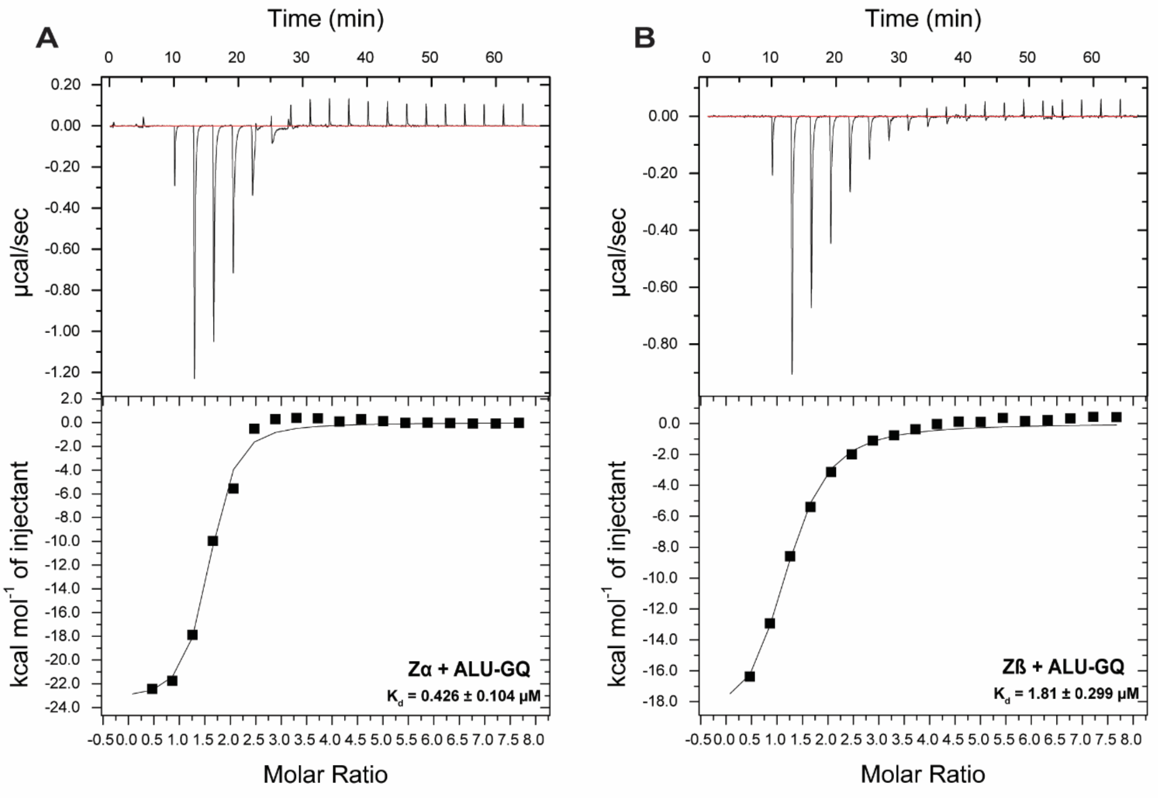

Figure S20: **Full isothermal titration calorimetry spectra of ADAR1 ZBDs binding to ALU-GQ$_{RNA}$.** A) Zα domain + ALU-GQ$_{RNA}$. B) Zβ domain + ALU-GQ$_{RNA}$.

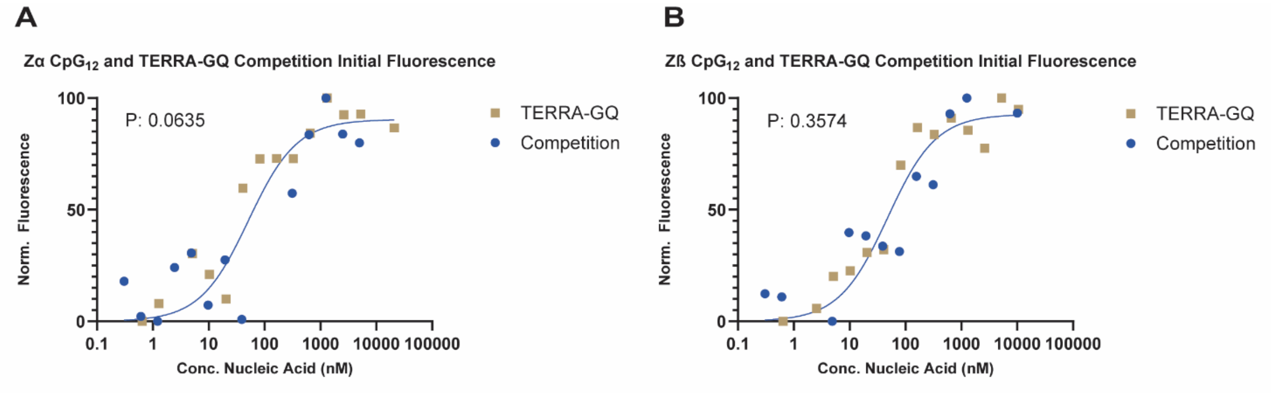

Figure S21: **Competition assay between TERRA-GQ$_{RNA}$ and Z-prone dsRNA binding to Zα or Zβ of ADAR1.** Binding curves titrating TERRA-GQ$_{RNA}$ in the presence of constant $(CpG)_{12}$ RNA for either A) ADAR1 Zα and B) ADAR1 Zβ. Zα or Zβ was at a concentration of 40 nM and $(CpG)_{12}$ was added to a concentration of 500 nM after labelling and incubated at 42 °C for 30 minutes prior to MST measurement.

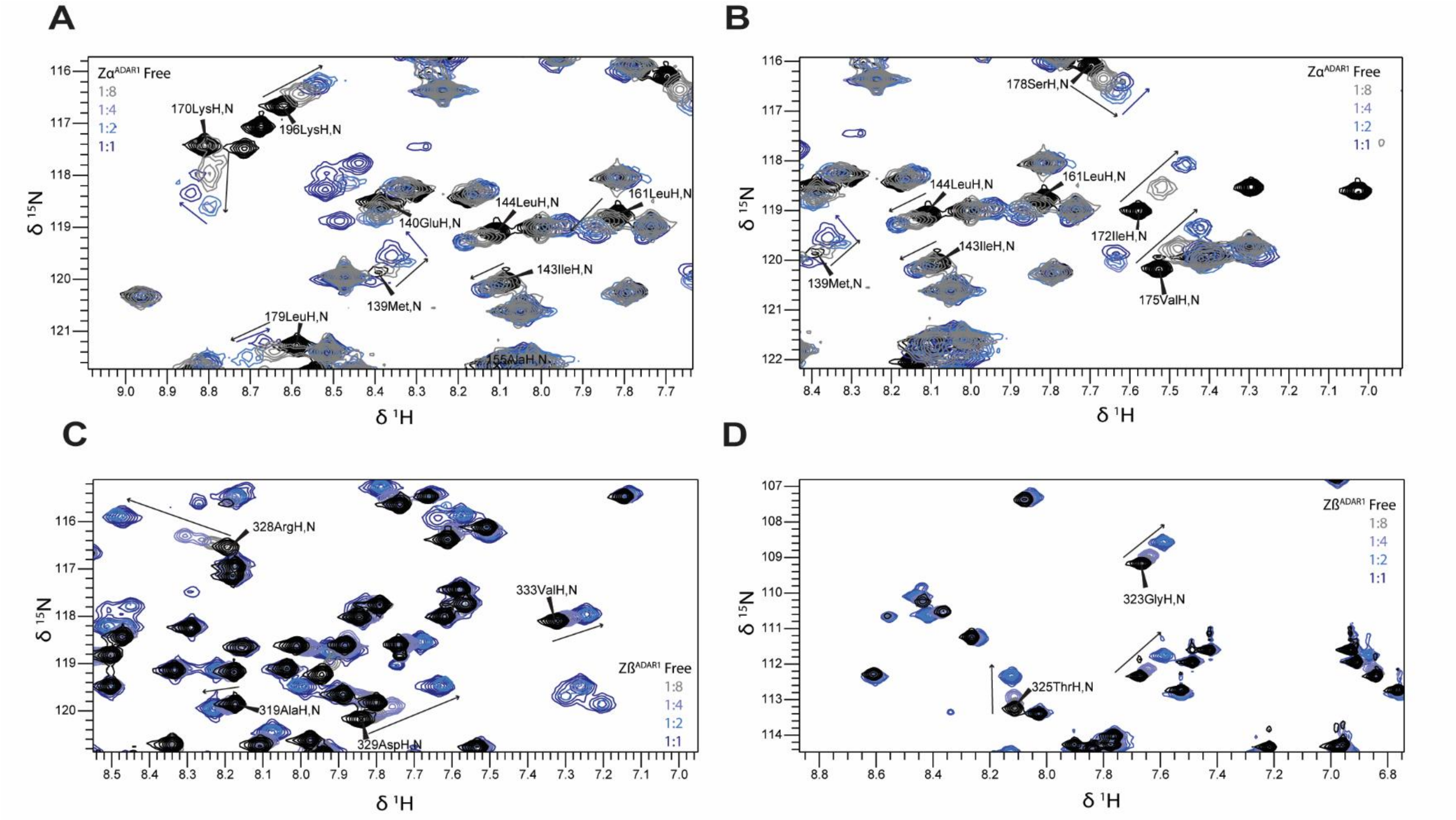

Figure S22: **ADAR1 Zα and Zβ titration series with TERRA-mut$_{RNA}$.** A, B) Extended regions of overlaid HSQC spectra of Zα domain with increasing concentrations of TERRA-mut$_{RNA}$. Concentrations are 200 μM Zα, and 25, 50, 100, and 200 μM TERRA-mut$_{RNA}$. C, D) Extended regions of overlaid HSQC spectra of Zβ domain with increasing concentrations of TERRA-mut$_{RNA}$. Concentrations are 200 μM Zβ, and 25, 50, 100, and 200 μM TERRA-mut$_{RNA}$.

3 NMR confirms the nucleic acid binding preference of the Zα and Zβ domains

Despite extensive peak disappearance in the HSQC spectra of the ADAR1 ZBDs in complex with many constructs (Zα with TERRA-GQ_{RNA}, Zβ with ALU-GQ_{RNA}, U-loop-GQ_{RNA}, TERRA-GQ_{RNA}), other constructs instead show CSPs with varying degrees of peak shifts. For Zα, we obtained measurable CSPs for both mutant sequences and the TTT-loop-GQ_{DNA}, as well as minor but consistent CSPs for U-loop-GQ_{RNA}. Notably, certain residues consistently shifted in the same direction upon the addition of nucleic acid (Fig. S23A). This consistent directionality and ordering of CSPs suggest that these are residues that specifically interact with these nucleic acids, providing a means to investigate Zα's preference across constructs. Comparing these CSPs to titration curves with TERRA-mut_{RNA}, which show similar directional shifts as a function of ligand concentration, supports the hypothesis that for each construct, peak shift magnitude is likely correlated with the binding strength (Fig. S22).

When comparing NA constructs in interaction with Zα, a trend emerges: the two non-GQ constructs (TERRA-mut_{RNA} and ALU-mut_{RNA}) consistently exhibit the greatest magnitude of CSPs, whereas the two GQ constructs with visible CSPs (TTT-loop-GQ_{DNA} and U-loop-GQ_{RNA}) exhibit smaller changes (Figs. S23A, B). This suggests that Zα preferentially binds non-GQ constructs over these specific GQs, particularly TTT-loop-GQ_{RNA}. However, certain residues deviate from this trend: some display CSPs for TTT-loop-GQ_{DNA} but not for the other three constructs or shift in a different direction than observed for the other sequences (residues 157, 162) (Fig. S23B). This may reflect residues in Zα that interact differently with GQ than with non-GQ constructs, mediate specific DNA interactions, or be due to the fact that TTT-loop-GQ_{DNA} is the only anti-parallel GQ in this study. These trends are consistent with our affinity measurements, in which TERRA-mut_{RNA} yields a tighter affinity than these GQs, while Zα interacts weakest with the TTT-loop-GQ_{DNA}.

Some GQ constructs retained sufficient peak intensity after addition of Zβ to analyze CSPs. We compared TERRA-mut_{RNA}, ALU-mut_{RNA}, TTT-loop-GQ_{DNA} and TERRA-GQ_{RNA}. Once again, a consistent trend emerged here: TERRA-GQ_{RNA} peaks always shifted further than or equal to the other constructs, in accordance with its tight affinity measured via MST. TTT-loop-GQ_{DNA} CSPs were next, followed by those of TERRA-mut_{RNA}, and finally ALU-mut_{RNA} showed the least (Figs. S23C, D). Zβ exhibited greater CSPs when binding TTT-loop-GQ_{DNA} than any Zα. This could be a result of either a greater DNA or antiparallel GQ binding ability, potentially reflecting a unique biological role. These results further confirm both the affinity measurements as well as the 6mer HSQC spectra, indicating that Zβ, but not Zα, has GQ structural specificity, as well as tighter binding of Zβ to specific GQs than Zα.

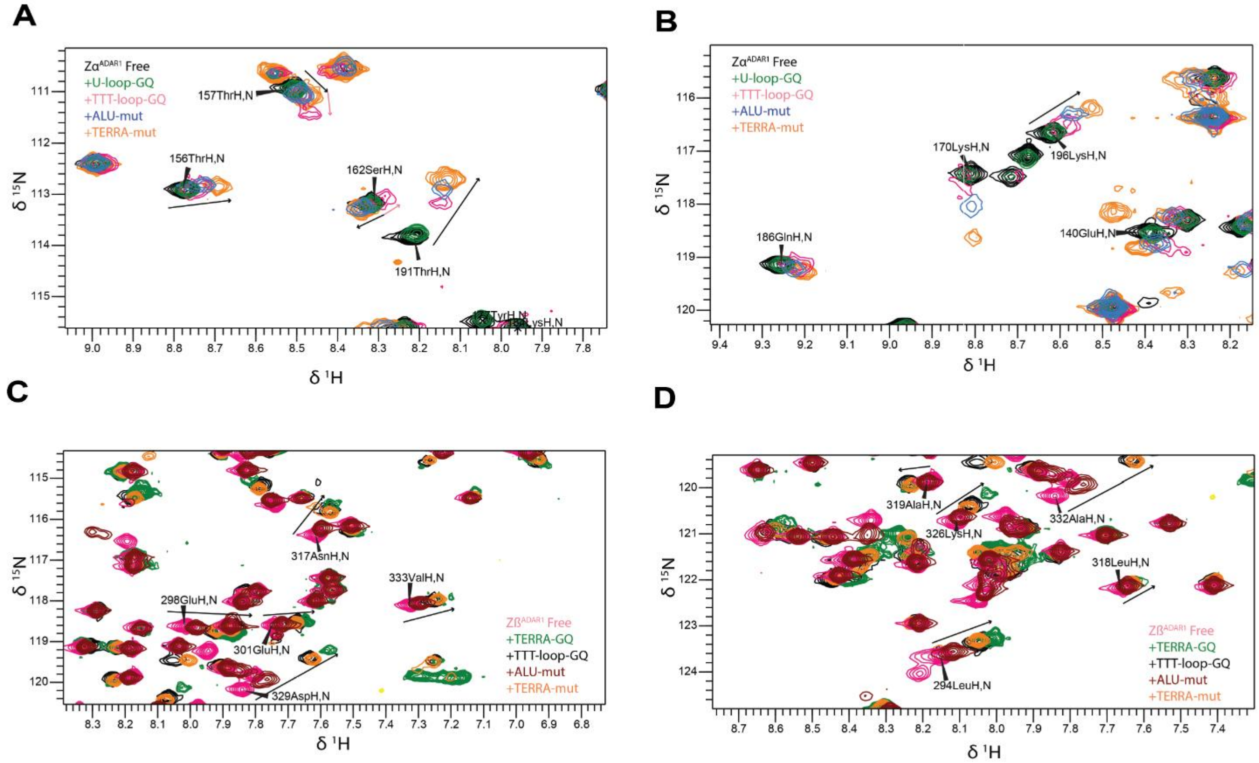

Figure S23: **DNA and RNA constructs induce varying peak shift changes in ADAR1 Zα and Zβ.** A, B) Extended regions of overlaid HSQC spectra of free Zα and in complex with TERRA-mut$_{RNA}$, ALU-mut$_{RNA}$, TTT-loop-G$_{DNA}$, and U-loop-G$_{RNA}$ are shown. C, D) Extended regions of overlaid HSQC spectra of free Zα and in complex with TERRA-mut$_{RNA}$, ALU-mut$_{RNA}$, TTT-loop-G$_{DNA}$, and U-loop-G$_{RNA}$ are shown.

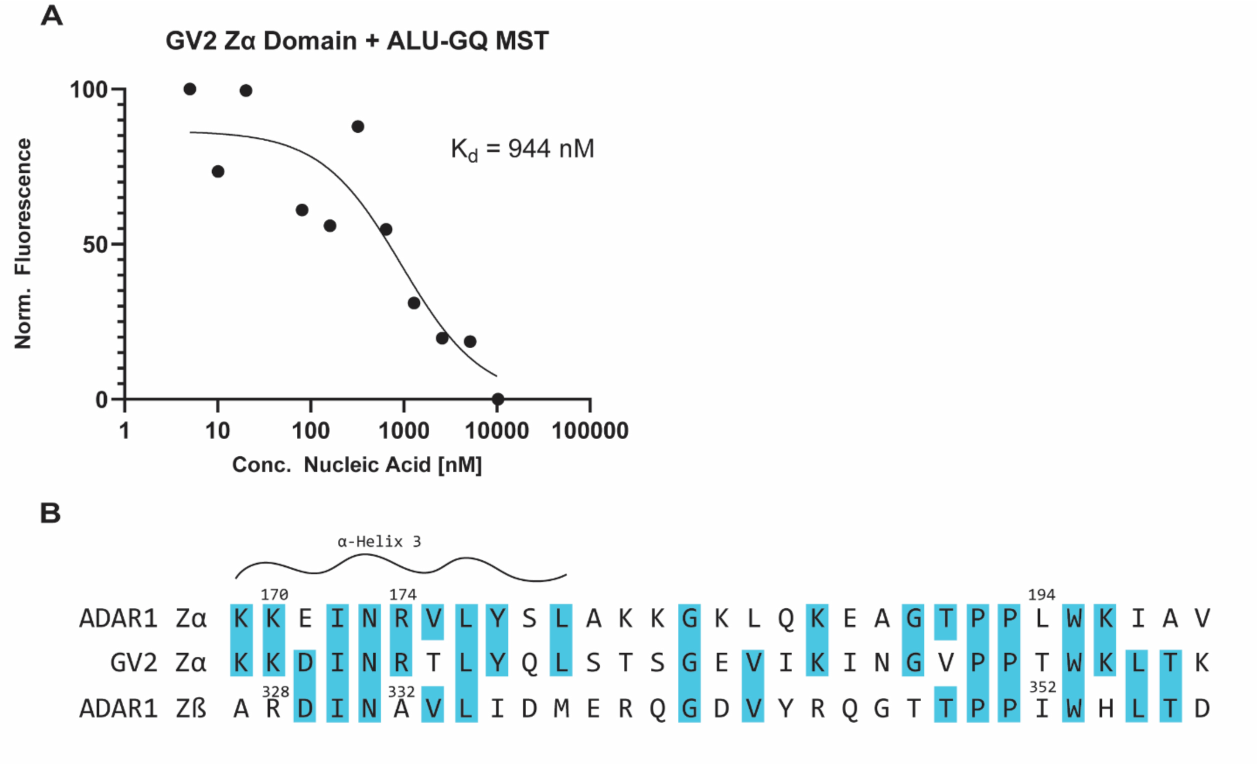

Figure S24: **A viral Zα domain binds to ALU-GQ$_{RNA}$.** A) 1.5 s MST curve fit of GV2 Zα with ALU-GQ$_{RNA}$. B) Sequence alignment of ADAR1 Zα, Zβ [uniprot: p55265], and GV2 Zα.

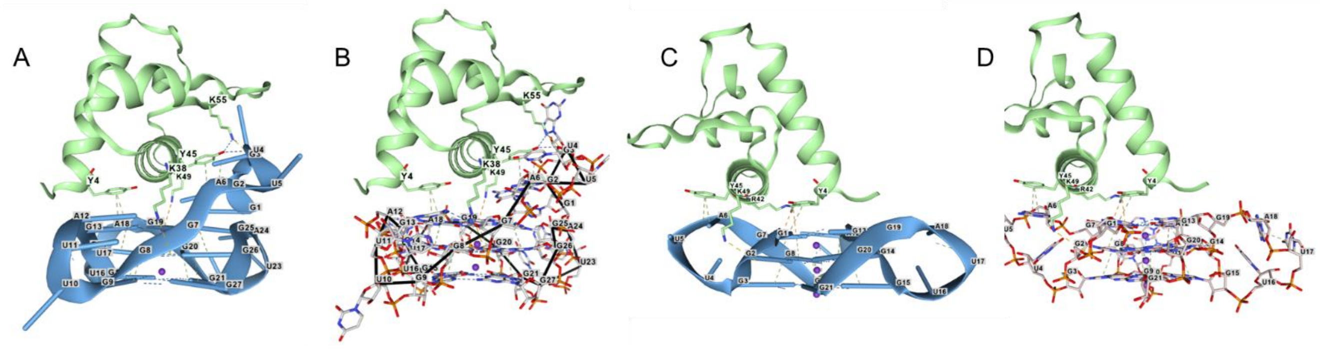

Figure S25: **GQ variants and binding surfaces.** A, B) A “spare tire” G-quadruplex sequence $(GGGUUA)_4G_3$ was modeled with Zα as previously described[7]. The extra residues are incorporated into loop 1 and anchor additional interactions with residues in the α3 helix of the Zα fold. C, D) The interaction of Zα with a standard GQ under the same conditions, with fewer contacts stabilizing the interaction.

References

(1) del Villar-Guerra, R.; Trent, J. O.; Chaires, J. B. G-Quadruplex Secondary Structure from Circular Dichroism Spectroscopy. *Angew. Chem. Int. Ed Engl.* **2018**, *57* (24), 7171–7175. https://doi.org/10.1002/anie.201709184.

(2) Kypr, J.; Kejnovská, I.; Renčiuk, D.; Vorlíčková, M. Circular Dichroism and Conformational Polymorphism of DNA. *Nucleic Acids Res.* **2009**, *37* (6), 1713–1725. https://doi.org/10.1093/nar/gkp026.

(3) Luo, Y.; Živković, M. L.; Wang, J.; Ryneš, J.; Foldynová-Trantírková, S.; Trantírek, L.; Verga, D.; Mergny, J.-L. A Sodium/Potassium Switch for G4-Prone G/C-Rich Sequences. *Nucleic Acids Res.* **2024**, *52* (1), 448–461. https://doi.org/10.1093/nar/gkad1073.

(4) Mukundan, V. T.; Phan, A. T. Bulges in G-Quadruplexes: Broadening the Definition of G-Quadruplex-Forming Sequences. *J. Am. Chem. Soc.* **2013**, *135* (13), 5017–5028. https://doi.org/10.1021/ja310251r.

(5) Bhattacharyya, D.; Mirihana Arachchilage, G.; Basu, S. Metal Cations in G-Quadruplex Folding and Stability. *Front. Chem.* **2016**, *4*, 38. https://doi.org/10.3389/fchem.2016.00038.

(6) Nichols, P. J.; Krall, J. B.; Henen, M. A.; Welty, R.; Macfadden, A.; Vicens, Q.; Vögeli, B. Z-Form Adoption of Nucleic Acid Is a Multi-Step Process Which Proceeds through a Melted Intermediate. *J. Am. Chem. Soc.* **2024**, *146* (1), 677–694. https://doi.org/10.1021/jacs.3c10406.

(7) Herbert, A.; Cherednichenko, O.; Lybrand, T. P.; Egli, M.; Poptsova, M. Zα and Zβ Localize ADAR1 to Flipons That Modulate Innate Immunity, Alternative Splicing, and Nonsynonymous RNA Editing. *Int. J. Mol. Sci.* **2025**, *26* (6), 2422. https://doi.org/10.3390/ijms26062422.
